# Extracellular matrix profiles determine risk and prognosis of the squamous cell carcinoma subtype of non-small cell lung carcinoma

**DOI:** 10.1186/s13073-022-01127-6

**Published:** 2022-11-21

**Authors:** Amelia L. Parker, Elise Bowman, Adriana Zingone, Brid M. Ryan, Wendy A. Cooper, Maija Kohonen-Corish, Curtis C. Harris, Thomas R. Cox

**Affiliations:** 1 Matrix and Metastasis Lab, Cancer Ecosystems Program, Garvan Institute of Medical Research and The Kinghorn Cancer Centre, 384 Victoria St, Darlinghurst, NSW 2052 Australia; 2 School of Clinical Medicine, UNSW Sydney, Sydney, 2052 Australia; 3 Laboratory of Human Carcinogenesis, Center for Cancer Research, National Cancer Institute, Bethesda, MD 20892 USA; 4 Present address: MiNA Therapeutics, London, UK; 5 Department of Tissue Pathology and Diagnostic Oncology, NSW Health Pathology, Royal Prince Alfred Hospital, Camperdown, NSW 2050 Australia; 6 Sydney Medical School, University of Sydney, Sydney, NSW 2050 Australia; 7 Discipline of Pathology, School of Medicine, Western Sydney University, Liverpool, NSW 2170 Australia; 8 Woolcock Institute of Medical Research, Sydney, NSW 2037 Australia; 9 Microbiome Research Centre, School of Clinical Medicine, UNSW Sydney, Sydney, 2052 Australia; 10 Garvan Institute of Medical Research, Darlinghurst, NSW 2010 Australia

**Keywords:** Extracellular matrix, Non-small cell lung cancer, Squamous

## Abstract

**Background:**

Squamous cell carcinoma (SqCC) is a subtype of non-small cell lung cancer for which patient prognosis remains poor. The extracellular matrix (ECM) is critical in regulating cell behavior; however, its importance in tumor aggressiveness remains to be comprehensively characterized.

**Methods:**

Multi-omics data of SqCC human tumor specimens was combined to characterize ECM features associated with initiation and recurrence. Penalized logistic regression was used to define a matrix risk signature for SqCC tumors and its performance across a panel of tumor types and in SqCC premalignant lesions was evaluated. Consensus clustering was used to define prognostic matreotypes for SqCC tumors. Matreotype-specific tumor biology was defined by integration of bulk RNAseq with scRNAseq data, cell type deconvolution, analysis of ligand-receptor interactions and enriched biological pathways, and through cross comparison of matreotype expression profiles with aging and idiopathic pulmonary fibrosis lung profiles.

**Results:**

This analysis revealed subtype-specific ECM signatures associated with tumor initiation that were predictive of premalignant progression. We identified an ECM-enriched tumor subtype associated with the poorest prognosis. In silico analysis indicates that matrix remodeling programs differentially activate intracellular signaling in tumor and stromal cells to reinforce matrix remodeling associated with resistance and progression. The matrix subtype with the poorest prognosis resembles ECM remodeling in idiopathic pulmonary fibrosis and may represent a field of cancerization associated with elevated cancer risk.

**Conclusions:**

Collectively, this analysis defines matrix-driven features of poor prognosis to inform precision medicine prevention and treatment strategies towards improving SqCC patient outcome.

**Supplementary Information:**

The online version contains supplementary material available at 10.1186/s13073-022-01127-6.

## Background

The squamous subtype of non-small cell lung cancer (NSCLC) is the second most common lung cancer subtype [1, 2], contributing to a lung cancer survival rate of only 20.2% [3]. The lack of actionable mutations in this cancer type has hindered the development of precision medicine protocols that have substantially improved patient survival in subgroups of adenocarcinoma patients [4]. Platinum doublet therapy remains the first-line treatment for the majority of SqCC patients; however, it is not yet possible to predict which patients will respond. In early-stage patients, where surgery is the treatment of choice and potentially curable, up to 30% will have their disease recur [5].

Worldwide, lung cancer screening programs have demonstrated substantial improvements in patient mortality through early detection and intervention [6]. The increased detection of early, pre-invasive lesions also raises clinical challenges as 30% of pre-invasive lesions will spontaneously regress in the absence of intervention [7, 8]. There is currently no way of identifying which premalignant lesions will become invasive and progress to cancer. Robust identification of individuals at high risk of premalignant progression will be an important keystone for the successful implementation of screening strategies for lung cancer patients globally.

The high degree of subclonal tumor heterogeneity [9] and high mutational burden [10] in SqCC tumors make it difficult to achieve sustained clinical responses when treating cancer cells alone. This has been illustrated in recent precision medicine trials targeting specific cancer cell mutations in advanced stage patients who were ineligible for surgery or radiotherapy or those who had previously failed platinum doublet therapy, where only a small proportion of patients demonstrated clinical responses [11, 12]. The tumor microenvironment is increasingly acknowledged to play an important role in regulating the clonal evolution of the tumor, and the sensitivity of cancer cells to both chemotherapy and immunotherapies. Evidence in other cancer types indicates that the cell extrinsic factors from the tumor microenvironment have the potential to override cancer cell intrinsic signaling to regulate tumor progression independently of the clonal heterogeneity of the tumor [13]. Complementary treatment approaches that target the tumor-supporting components of the local microenvironment have the potential to simultaneously target multiple cancer cell clones to achieve more durable clinical responses in these highly heterogeneous tumors.

Fundamental to the success of a co-targeting approach is the ability to identify prognostic features of SqCC tumors and exploit these vulnerabilities for therapeutic benefit. A number of approaches have been employed to develop prognostic signatures in lung cancer, with the majority being developed in adenocarcinoma subtypes only [14–16], or of mixed NSCLC subtypes including both adenocarcinoma and squamous NSCLC [17–20]. Those developed for SqCC specifically have been developed by genome wide profiling and show little overlap [21–23]. Molecular subtyping of SqCC tumors has revealed distinct and heterogeneous SqCC tumor types at the transcript and protein level that are characterized by varying degrees of immunological activation, proliferation, and differentiation [24, 25]. Importantly, these appear to have a distinct biology from lung adenocarcinomas [26–28], suggesting that prognostic programs operate differently between these major NSCLC subtypes. Improvements in SqCC treatment will require a thorough understanding of how SqCC biology differs from adenocarcinoma, and identification of therapies that may be more effective in this specific subtype.

The extracellular matrix (ECM) is an important regulator of cell behavior [29, 30] and is increasingly recognized to regulate tumor progression in multiple cancer types [31]. The ECM and its remodeling by cancer, stromal and immune cells have been shown to promote the growth, survival, and metastasis of cancer cells [32–34]. Associations between ECM expression and prognosis have been identified in NSCLC generally [17] and the expression and prognostic value of specific ECM proteins, such as tenascin-C [14], have been more extensively studied in adenocarcinoma than squamous NSCLC [35]. In SqCC tumors, for example, higher expression of the glycoprotein periostin was observed compared with adenocarcinomas, where it is expressed by activated fibroblasts [36] and is associated with survival [36, 37]. In addition, the glycoproteins thrombospondin 1 and 2 are overexpressed in SqCC [38]. However, it remains unclear how the SqCC ECM environment differs from that of the normal lung, and if ECM remodeling can inform diagnostic and therapeutic strategies in this poor-prognosis cancer. In addition, matrix molecules do not operate in isolation, but rather as integrated macromolecular networks [31]. A broad perspective of how these matrix networks are modulated in SqCC tumors, and the impact of this on cancer cell behavior is needed. The approval of the anti-stromal therapy nintedanib as a second-line therapy in adenocarcinoma patients [39] and clinical responses in Phase I trials in SqCC patients [40] attests to the potential for a co-targeting approach in the treatment of the lung cancer more generally. A more extensive examination of the ECM, and the role it plays in SqCC would be fundamental to the effective implementation of matrix co-targeting approaches specifically in this NSCLC subtype.

Here we present an unbiased examination of bulk and single-cell transcriptomic and proteomic analysis to examine the ECM landscape in SqCC, and to define matrix-associated signatures of risk and prognosis. Overlapping ECM remodeling with aging and chronic lung diseases suggests that early-stage changes in the lung ECM may promote tumor initiation and contribute to the increased incidence of lung tumors with age. Prognostic ECM subtypes (matreotypes [41]) identified in this study suggest that a subset of poor-prognosis SqCC patients may benefit from the repurposing of existing stromal therapies as part of a co-targeting approach.

## Methods

### Clinical samples

Publicly available data was obtained as described (Table 1) and analyzed in accordance with the respective guidelines of each platform. TCGA data (RNA sequencing, whole exome sequencing, copy number variation, and reverse phase protein array data) from 223 SqCC patients (including 17 patients with matched non-involved non-tumor tissue) and 162 adenocarcinoma patients (including 37 patients with matched non-involved non-tumor lung tissue) were obtained as described in Table 1 for assessment of ECM gene expression, associated tumor biology, and patient outcome. SqCC lung tumor and non-tumor biospecimens from the NCI-MD cohort (30 patients from the greater Baltimore area with stage I-III tumors) were collected and processed for RNA sequencing and analyzed for associations with patient outcome according to approved procedures as described previously [52, 53] (National Cancer Institute, USA, IRB OH98-C-N027). SqCC tissue microarrays were generated from primary lung SqCC samples obtained from 94 patients with stage I–III tumors resected at Royal Prince Alfred Hospital, Sydney, NSW, Australia, between 1996 and 2002 as described previously [54]. Briefly, each core was 1mm in diameter and consisted of 3–6 tumor core replicates. These tissue microarrays were stained with picrosirius red, imaged using polarized microscopy and analyzed for associations with patient survival according to approved protocols (Royal Prince Alfred Hospital and Garvan Institute of Medical Research, HREC10/RPAH/491, X14-0359, Garvan GHRP 1423). SqCC scRNAseq data was obtained from Lambrechts et al. (see Table 1) derived from 3 patients who had undergone lobe resection as previously described [46]. The clinicodemographic features of these cohorts are outlined in Supplementary Tables 4, 5 and 6. Written informed consent was received prior to participation in these studies.

**Table 1:** Datasets accessed in this study

**Datasets**			
**Cohort**	**Data type**	**Source**	**Reference**
*TCGA LUSC*	RNAseq, WES, CNV, RPPA (Level 4 data)	GDAC Firehose, TCPA Portal	[42, 43]
*TCGA LUAD*	RNAseq	GDAC Firehose	[44]
*NCI-MD Cohort SqCC*	RNAseq	This study; Gene Expression Omnibus, GSE201221	
*UHN Cohort*	Microarray	Gene Expression Omnibus, GSE50081	[20]
*TCGA Pan-Cancer Cohort*	RNAseq	https://gdc.cancer.gov/about-data/publications/pancanatlas with survival data from [45]	[45]
*SqCC cell types from scRNAseq*	scRNAseq	Loom files and Signature Matrix	[46]
*Premalignant lesions*	RNAseq	Gene Expression Omnibus GSE108124	[7]
*Aging lung*	RNAseq	Gene Expression Omnibus GSE165192	[47]
**Data and signatures**			
**Data type**	**Data format**	**Source**	**Reference**
*IPF fibrosis score*	Expression Data	Manuscript	[48]
IPF fibroblast phenotypes from scRNAseq	scRNAseq	Gene Expression Omnibus	[49]
*IPF cell types from scRNAseq*	Signature matrix	Manuscript	[50]
*Lung cancer-specific immunological cell types*	Gene Signatures	Manuscript	[51]

### RNA sequencing

Lung tumor and adjacent non-tumor tissue samples were collected as part of the NCI- University of Maryland Study according to protocols approved by the institutional review board (OH98-C-N027, National Cancer Institute, USA) and processed as described previously [53, 55] and outlined in detail in Supplementary Methods. Gene expression levels were quantified using RSEM [56] and batch corrected (Combat algorithm, SVA package [57]). Additional details are provided in Supplementary Methods.

### Bulk RNAseq analysis of squamous cell carcinomas

RSEM-quantified RNAseq abundances and relevant clinical information from publicly available datasets were accessed according to Table 1. Gene level counts were filtered and TMM normalized (EdgeR package [58]) before being log_2_ transformed.

Matrisomal genes were defined as core matrisome or matrisome-associated according to Naba et al. [29]. Principal component analysis of tumor compared with non-tumor tissue was performed using the prcomp algorithm and default settings in base R. Differential gene expression analysis between tumor and non-tumor tissue was performed using the Limma package [59]. Heatmaps of gene expression matrices were generated using the ComplexHeatmap package [60] for visualization. Additional details are provided in Supplementary Methods.

### Proteomic analysis of squamous cell carcinomas

The proteome of TCGA SqCC samples were analyzed using the Level 4 Reverse Phase Proteomic Array (RPPA) data downloaded from the TCPA portal (https://tcpaportal.org/tcpa/download.html) [42]. Differential enrichment of proteomic data between ECM-High and ECM-Low Matreotypes was performed using Mann-Whitney *U* tests with Benjamini-Hochberg correction for multiple comparisons.

### Tumor purity analysis

To assess the tumor purity and relative stromal and immune composition of bulk RNAseq data from the TCGA LUSC dataset, ESTIMATE scores of tumor purity, including stromal and immune admixture scores [61] for each sample were accessed from the MD Anderson Bioinformatics server (https://bioinformatics.mdanderson.org/estimate/disease.html) as the LUSC samples for RNAseqv2 data.

### Squamous cell carcinomas canonical molecular subtypes

Canonical SqCC molecular subtypes (Basal, Classical, Primitive, Secretory) defined by Wilkerson et al. [24] were assigned to TCGA LUSC bulk RNAseq samples as the canonical molecular subtype with the highest Pearson correlation coefficient using the published centroids [24]. Samples were classified as primitive (9.4%), classical (42.1%), secretory (21.5%), and basal (26.9%), a distribution that is consistent with previous reports [24, 43]. Enrichment of canonical molecular subtypes within ECM-High and ECM-Low matreotypes was assessed by Fisher’s exact test.

### Matrix risk signature generation

Risk signature feature selection was performed on differentially expressed core matrisomal genes between tumor and non-tumor tissue in the TCGA LUSC dataset. Genes were selected based on their association with tumor compared with non-tumor tissue using Elastic Net penalized logistic regression (glmfit algorithm, glmnet package with alpha =0.5 [62]), to account for the high degree of correlation of some matrix genes. *Z*-scaled RNAseq expression data was initially partitioned 80% to 20% into training and test datasets, respectively (createDataPartition algorithm, caret package) and the model was developed using training data only. The coefficient of shrinkage (lambda) was chosen as the value within one standard error of the lambda that minimized the cross-validation prediction error rate (cv.glmnet algorithm, glmnet package). To generate a matrix risk score and for visualization purposes, odds ratios were calculated using Firth’s correction (logistf algorithm, logistf package). The matrix risk score for each sample was summed as the product of the log(odds ratio) (Supplementary Table 1) and the expression value for each *z*-scaled gene expression (Equation 1). Additional details are provided in Supplementary Methods.

1 $$Matrix\ Risk\ Score={\sum}_{i=1}^n{z}_i{\beta}_i$$

where *i* = gene in the matrix risk signature of length n

*z*_*i*_ = *z*-scaled gene expression of gene *i*

*β*_*i*_ = log (odds ratio) of gene *i*

### Matreotype identification

Identification of matreotypes was performed using Monte-Carlo reference-based consensus clustering (M3C command using the K-means clustering algorithm with default settings, M3C package [63]) applied to the TCGA expression matrix of significantly differentially expressed core matrisomal genes from tumor and non-tumor tissue. Cluster number was determined to give a maximal Relative Cluster Stability Index (RCSI), a Monte-Carlo *p*-value less than 0.05 and a minimal Proportional of Ambiguous Clustering (PAC) Score [63].

Centroids for each matreotype were calculated on the TCGA LUSC dataset as the mean *z*-scaled expression level for significantly differentially expressed core matrisomal genes between tumor and non-tumor tissue. Matreotypes were assigned as the minimum Euclidean distance between each sample and the matreotype centroids. The association of matreotypes with categorical clinicodemographic information (Fisher’s exact test) and survival (survival and survminer packages) was assessed. Hazard ratios were calculated (coxph function, survminer package) with corrections for age and stage, which were clinical covariates significantly associated with outcome in univariate analyses. Gender, smoking status, and pack years were not statistically significantly associated with outcome in univariate analyses.

Matreotype associations with driver mutations were assessed using maftools. FGFR copy number variation analysis was performed on TCGA CNV data using probes mapped to the segment containing the FGFR cluster at chromosome 8p11.23 (Hg18; 38 387 813-38 445 509) with segment means < −0.7 and > 0.7 defining copy number losses and gains, respectively. The association of FGFR amplifications with ECM-High and ECM-Low matreotypes was assessed by Fisher’s exact test. Additional details are provided in Supplementary Methods.

### Pathway analyses

Pathway analysis was performed on the 50 hallmark pathways and C2 oncogenic pathways described in the molecular signatures database (mSigDb package (https://davislaboratory.github.io/msigdb)). Pathway activity estimates were applied to each sample (gsva algorithm with default settings, GSVA package [64]) and differential pathway enrichment was assessed using Limma [59] as described in detail in the Supplementary Methods.

### Ligand-receptor interaction analysis

Genes within the RNAseq datasets were annotated as ligands and receptors based on the curated database of human ligand-receptor pairs previously published by Ramilowski et al. [65] based on supporting literature. Only ligands corresponding to core matrisome genes as defined by Naba et al. [29] were retained for further analysis. The strength of the interaction between core matrisomal genes and their receptors were calculated as the product of expression values from the ligand (i.e., core matrisomal gene) and its cognate receptor in each sample (Equation 2), as described previously [66].

2 $$Interaction\ Score={R}_i{L}_i$$

where *R*_*i*_ = *z*-scaled gene expression of receptor *i*

*L*_*i*_ = *z*-scaled gene expression of ligand *i*

Receptors were then grouped into receptor classes, and interaction scores for each receptor class were calculated as the maximum interaction score for that receptor class. Receptors were also mapped to their corresponding mSigDb Hallmark pathways.

Differential enrichment of ligand-receptor interaction scores or ligand-pathway interaction scores in the different matreotypes were tested using Limma (limma package) [59] and visualized as a circos plot (circos algorithm, circlize package [67]). Additional detail is provided in Supplementary Methods.

### Cellular composition analysis

Two main approaches were implemented to identify enriched cell types in SqCC matreotypes using datasets described in Table 1. Deconvolution of bulk TCGA LUSC RNAseq data into NSCLC epithelial, fibroblast, and endothelial cell types was performed using the signature matrix derived from scRNAseq analysis of NSCLC tumors published by Lambrechts et al. [46] and CibersortX (absolute mode, batch correction, 100 permutations). Differential enrichment of cell types was tested using the limma package [59].

The relative enrichment of immune cells within the tumor microenvironment was assigned to each sample by applying NSCLC-specific immune cell signatures developed by Faruki et al. [51] using Gene Set Variation Analysis (gsva algorithm, GSVA package, default settings). Differential enrichment of immune cell type scores in the ECM-High vs ECM-Low matreotypes were assessed by Kruskal-Wallis test with Benjamini-Hochberg multiple comparisons correction. Additional detail is provided in Supplementary Methods.

### Single-cell RNAseq analysis of squamous cell carcinoma

Raw gene expression matrices and cellular metadata including cell type assignments from scRNAseq data of NSCLC samples were obtained and processed as described [46, 68] (Seurat v4.0.1; see Supplementary Methods for details). Cell type scores for ECM genes were assigned using the AddModuleScore function (Seurat package, default settings). Matrix risk scores were calculated on the *z*-scaled gene expression matrix for each cell type using Equation 1 as described above (Matrix Risk Signature section).

### Fibrosis score

A fibrosis score was calculated based on the idiopathic pulmonary fibrosis signature described by McDonough et al. [48] (see Supplementary Methods). Briefly, normalized gene expression was weighted in the positive and negative direction for genes up- and downregulated in IPF lungs, respectively (Equation 3).

3 $$IPF\ Fibrosis\ Score={\sum}_i^n{w}_i{z}_i$$

where *w*_*i*_ = 1 for gene upregulated in IPF lungs and −1 for genes downregulated in IPF lungs for gene *i* in the *n* gene signature

*z*_*i*_ = scaled gene expression for gene *i* in the *n* gene signature

### Transcription factor enrichment analysis

Transcription factor analysis on core matrisomal correlation clusters was performed on gene lists corresponding to each correlation cluster using the chEA transcription factor targets database and chEA3 web interface [69] (see Supplementary Methods for details). The integrated mean rank for each transcription factor was calculated for each correlation cluster.

### Picrosirius red staining and quantitation in tissue microarray

Picrosirius red staining of SqCC TMAs was performed as described previously [70]. Stained sections were imaged on a Leica DMI 6000 fitted with posterior and anterior polarizing filters and picrosirius red signal relative to tissue area was quantified using an in-house script published previously [70]. The maximum picrosirius red signal over three to five cores was calculated per patient. The association of picrosirius red signal with survival was performed using Cox proportional hazards models (coxph algorithm and survival package). See Supplementary Methods for additional detail.

### Statistics

All statistical analysis was performed in R (v3.6.3) and GraphPad Prism (v8). *P*-values were adjusted for multiple comparisons using the Benjamini-Hochberg method. Statistical testing of two groups was performed by the non-parametric Mann-Whitney *U* test and of more than two groups by the Kruskal-Wallis test. *P*-values less than 0.05 were considered significant.

### Data and code availability

Publicly available data used in this study are accessible as listed in Table 1. The NCI-MD cohort RNAseq data analyzed in this study are accessible at the NCBI GEO website under the accession number GSE201221 (https://www.ncbi.nlm.nih.gov/geo/query/acc.cgi?acc=GSE201221) [71]. Publicly available TCGA LUSC and LUAD RNAseq, WES, and CNV data are available from Broad GDAC Firehose (https://gdac.broadinstitute.org). Publicly available Pan-Cancer RNAseq and survival data is available from Genomic Data Commons repository (https://gdc.cancer.gov/about-data/publications/pancanatlas).

Publicly available TCGA RPPA data is available from the TCPA Portal (https://tcpaportal.org/tcpa/download.html). Publicly available data from the UHN cohort is available in the Gene Expression Omnibus repository, GSE50081 (https://www.ncbi.nlm.nih.gov/geo/query/acc.cgi?acc=GSE50081). Publicly available RNAseq of squamous carcinoma in situ is available in the Gene Expression Omnibus repository, GSE108124 (https://www.ncbi.nlm.nih.gov/geo/query/acc.cgi?acc=GSE108124). scRNAseq data are available from https://lambrechtslab.sites.vib.ve/en/data-access. RNAseq data of the aging lung is available in the Gene Expression Omnibus repository, GSE165192 (https://www.ncbi.nlm.nih.gov/geo/query/acc.cgi?acc=GSE165192). Code used in this analysis is available via GitHub (https://github.com/AParkerLab). All other relevant data supporting the conclusions of this article are included within the article and its supplementary information files. Additional information may be obtained from the corresponding authors upon reasonable request.

## Results

### Cellular heterogeneity contributes to tumor-specific ECM remodeling in squamous carcinoma

**Fig. 1:**
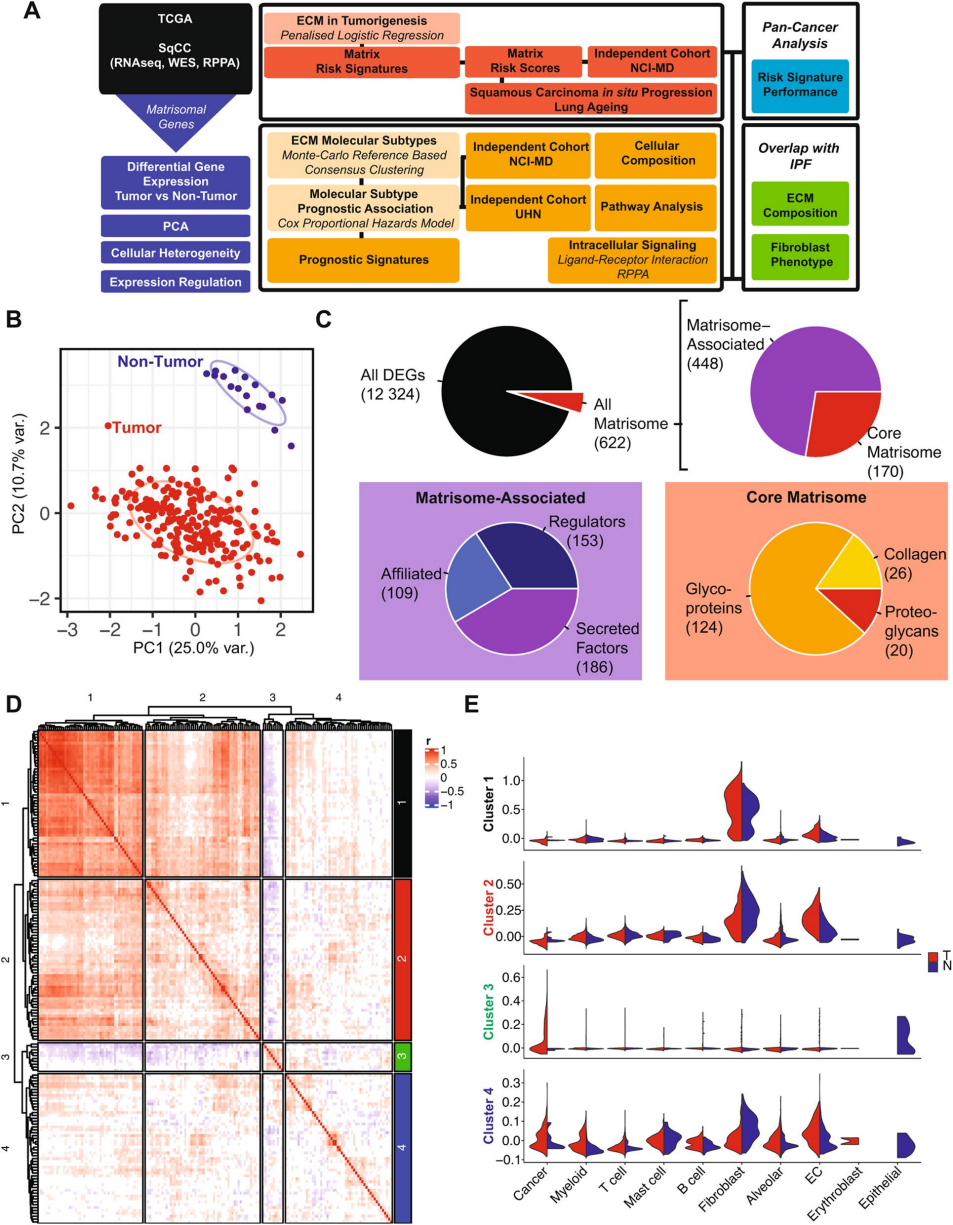
The ECM is significantly dysregulated in tumor compared with non-tumor tissue in SqCC. **A** Workflow describing the approach to characterizing the ECM landscape in SqCC. **B** Principal component analysis of core matrisomal gene expression in tumor (T) compared with non-tumor (NT) tissue. **C** Distribution of differentially expressed genes between tumor and non-tumor. 59.5% of matrisome-associated (488 genes) and 62.0% of core matrisomal (170) genes are differentially expressed in SqCC tumors. This represents 59.1% (26 genes), 57.1% (20 genes), and 63.6% (124 genes) of collagens, proteoglycans, and glycoproteins, respectively. It also represents 63.7% (109 genes), 64.3% (153 genes), and 54.1% (186 genes) of all ECM-affiliated proteins, ECM regulators, and secreted factors, respectively. Number of genes indicated in brackets. **D** Correlation analysis of differentially expressed core matrisomal genes identifies four major clusters of genes in SqCC tumors. r =Spearman correlation coefficient. Non-significant correlations are colored white; positively correlated genes are red and negatively correlated genes are blue. **E** Expression score of core matrisomal genes corresponding to the four correlational clusters shown in **D**, in specific lung cell types separated by tumor (T) and non-tumor (N) tissue. EC: endothelial cell

In order to comprehensively profile the ECM landscape in SqCC, we began by examining changes in the expression of ECM genes in tumor compared with non-tumor tissue using RNA sequencing data from The Cancer Genome Atlas [43] for which clinicodemographic and whole exome sequencing data was also available. Whole transcriptome RNA sequencing data from SqCC tumor (*n*=223) and matched, adjacent, non-involved non-tumor (*n*=17) tissues was filtered to include only matrisomal genes, as defined by Naba et al. [29] (Fig. 1A). These genes included 274 core matrisomal genes, which have clear structural roles in the ECM, as well as 753 matrisome-associated genes, which include enzymes involved in ECM remodeling and soluble factors that directly interact with the matrix [29].

Principal component analysis of core matrisomal gene expression indicated that the first two principal components of these genes were able to distinguish tumor from adjacent, non-involved lung tissue (Fig. 1B), suggesting that the expression of the core, structural components of the ECM in SqCC tumors substantially differs from that of normal lung.

Differential gene expression analysis of the global RNAseq data identified that 5.34% of all differentially expressed genes comparing tumor and non-tumor tissue were matrisomal genes (Fig. 1C). The majority of matrisomal genes were differentially expressed, with the expression of 62.0% of core and 59.5% of matrisome-associated genes differing between tumor and non-tumor tissue (Fig. 1C).

Comparative analysis with RNA sequencing data of 162 lung adenocarcinoma tissues (125 tumor, 37 non-tumor tissues) [44] revealed that 48.9% of all core matrisomal genes are differentially expressed between tumor and non-tumor tissues in both histological subtypes of NSCLC (Additional file 1: Fig. S1A). The majority of these genes were similarly regulated in adenocarcinoma tumors compared with matched non-tumor tissue suggesting that the dysregulation of many core matrisomal genes may be important in lung tumorigenesis in a subtype-independent manner (Additional file 1: Fig. S1B). However, the opposite expression of the basement membrane collagen *COL4A6*, basement membrane netrin *NTN5*, *VWA5B2*, and collagen fibrillogenesis regulator AEBP1 in SqCC compared with adenocarcinoma (Additional file 1: Fig. S1B) points to histological-subtype-specific changes in the core matrisome that may be important in tumor development. Further examination of collagen alpha-6 type IV (*COL4A6*) expression in tumor tissue for the two subtypes indicated that *COL4A6* expression did not significantly correlate with the expression of the adenocarcinoma marker *TT1* in adenocarcinoma (Additional file 1: Fig. S1C) but was significantly correlated with the SqCC marker *TP63* (Additional file 1: Fig. S1D), suggesting it is upregulated during squamous differentiation associated with tumor development in a cell-type-specific manner [72].

In recent years, it has become clear that single matrix molecules rarely function in isolation and instead function as part of a dynamic 3D supramolecular network of structurally and functionally integrated matrix components [31]. To understand whether core matrisomal genes were coordinately regulated in SqCC tumors, we performed correlation analysis on all differentially expressed core matrisomal genes in tumor tissues (Fig. 1D). Unsupervised hierarchical clustering of this analysis identified 4 major correlated core matrisomal clusters (Fig. 1D), broadly corresponding to a fibrotic ECM remodeling cluster (correlation cluster 1; including fibrillar collagens COL1A1, COL3A1, COL5A1, and pro-fibrotic glycoproteins THBS1 and THBS2); a basement membrane-enriched cluster (correlation cluster 2; including collagen type 4 (COL4A1, COL4A2, COL4A3, COL4A4, and basement membrane proteoglycan HSPG2); a glycoprotein-enriched cluster (correlation cluster 3; including BGLAP, ZP3, FNDC8, and EMILIN3); and with the fourth cluster consisting of genes which were not significantly correlated with one another (correlation cluster 4, including COL4A6, and CRELD1 and CRELD2 glycoproteins) (Fig. 1D). Correlated matrisomal gene expression profiles in bulk RNAseq may reflect coordinated regulation of gene expression within cells, differences in cell type composition among samples or combinations of both these scenarios. To understand in more detail how these correlated matrisomal genes may reflect the contributions of individual cells within the tumor microenvironment, we assessed the expression of these correlation clusters in individual cell types in tumor and non-tumor tissue from scRNAseq data of SqCC patients (3 patients with tumor and adjacent non-involved non-tumor tissue [46]). This analysis identified that the fibrotic correlation cluster genes (correlation cluster 1) were expressed largely by fibroblasts, while the basement membrane-enriched cluster genes (correlation cluster 2) also had additional contributions from endothelial cells (Fig. 1E, Additional file 1: Fig. S1E-H). The small group of glycoprotein-enriched genes (correlation cluster 3) were significantly expressed by epithelial cells in non-tumor tissue, and by cancer cells in tumor tissue (Fig. 1E, Additional file 1: Fig. S1I), indicating epithelial origin. Finally, the fourth cluster of genes (correlation cluster 4) was contributed to by multiple cell types within both the normal lung and tumor microenvironment (Fig. 1E, Additional file 1: Fig. S1J). Significant differences between tumor and non-tumor scores for fibroblasts, endothelial and epithelial cells in correlation clusters 1 to 3 (Fig. 1E) suggest that the presence of tumor cells likely induces altered matrisomal gene expression within stromal cells within the tumor microenvironment. Together, these analyses indicate that not only do cancer cells as well as resident stromal and immune cells within the SqCC tumor microenvironment contribute to significant ECM remodeling compared with normal lung tissue, but also that cellular heterogeneity within the tumor microenvironment contributes to, and likely underlies, the matrisomal profiles of SqCC tumors.

Co-regulation of gene transcription can also result in highly correlated gene expression networks. To examine this in more detail, we performed transcription factor enrichment analysis on the correlated gene clusters using chEA3 [69]. Because the transcriptional regulation of many ECM genes has not yet been comprehensively defined, integrating multiple CHIP databases with expression analysis in this approach expands the scope for identifying upstream regulators of matrix gene expression. This analysis identified enrichment of overlapping as well as distinct transcription factors for each cluster that have been implemented in lung development and cancer (Additional file 1: Fig. S1K). Consistent with the high correlation between genes in these clusters, correlation clusters 1 and 2 were both enriched with targets of AEBP1 and OSR1, which are expressed during fetal lung development [73] (Additional file 1: Fig. S1K). Conversely, correlation cluster 1 was selectively enriched for the EMT transcription factor TWIST2 while correlation cluster 2 was specifically enriched for FOXC2, which has been associated with mesenchymal development, and is known to be associated with platinum resistance and poor prognosis [74, 75]. Cluster 3 was enriched for LHX8 and FEV as well as DBX2, which is differentially expressed in hepatocellular carcinoma [76]. Finally, cluster 4 showed enrichment for SP7 and OLIG1, which has been previously shown to have prognostic value in NSCLC [77], as well as TP63, the master regulator of squamous differentiation [43] that correlates with *COL4A6* expression in this cluster (Additional file 1: Fig. S1D). While further experimental validation of these findings is required, these data highlight that common regulatory networks have the potential to co-regulate the expression of several matrix components within the tumor microenvironment. This, together with cell-type-specific matrix deposition from cancer cells, as well as normal and co-opted stromal and immune cells from the tumor microenvironment, is likely to contribute to dysregulated matrisomal gene expression in SqCC.

### ECM remodeling predicts squamous lung cancer risk

To more precisely define those ECM components that distinguish between tumor and non-tumor tissue in an unbiased manner, we further refined our list of differentially expressed matrisomal genes between tumor and non-tumor SqCC tissue using Elastic Net penalized logistic regression. This ensures that only the most significant genes were retained in the model while accommodating the high degree of correlation observed for some of the ECM genes (Fig. 1D). The final matrix risk model was then corrected for clinical covariates including age, sex, tumor stage, smoking status, and pack years [78] to minimize cohort bias.

**Fig. 2:**
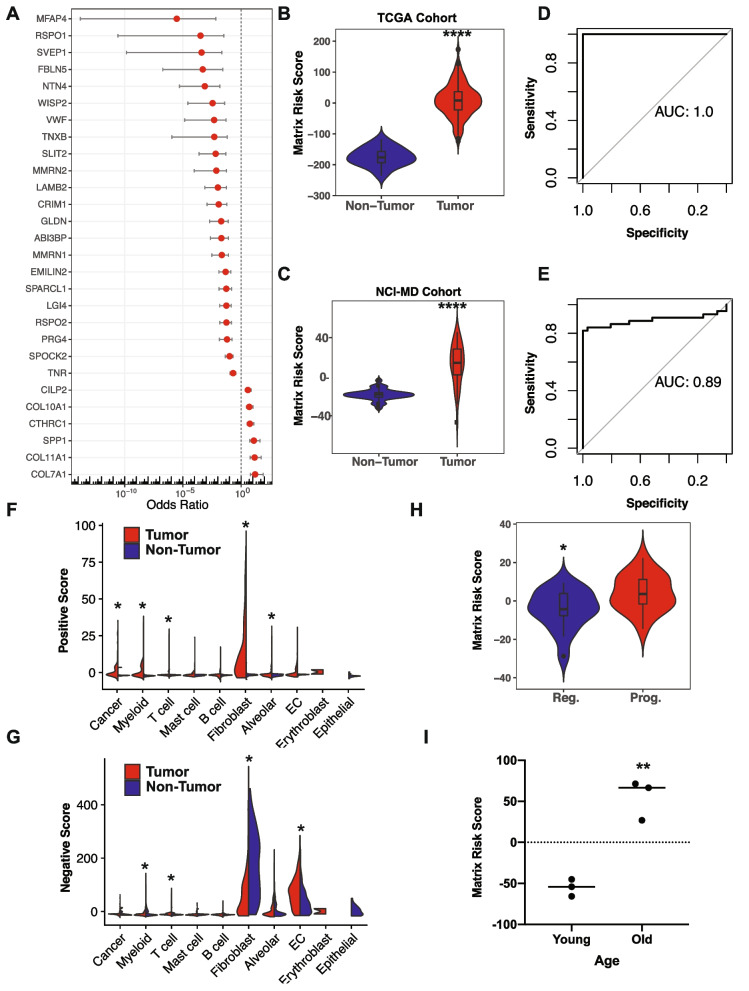
ECM changes associated with increased lung cancer risk and premalignant progression. **A** Odds ratios for genes in the matrix risk signature. See Additional File 1: Table S1. **B,C** Matrix risk scores are significantly higher in tumor compared with non-tumor tissue in the TCGA (**B**, *p*=6.7E−12, Mann-Whitney *U* test) and NCI-MD cohorts (**C**, *p*=4.6E−10, Mann-Whitney *U* test). **D,E** ROC analysis of the matrix risk score in distinguishing between tumor and non-tumor tissue on the bootstrapped test data from the TCGA cohort (**D**, area under the curve (AUC)=1) and the NCI-MD cohort (**E**, area under the curve (AUC) = 0.89). **F,G** Expression scores for matrix risk signature genes with positive odds ratios (**F**) and negative odds ratios (**G**) in specific lung cell types from tumor and non-tumor tissue. **H** Matrix risk score in regressive (Reg.) and progressive (Prog.) pre-invasive squamous carcinoma in situ lesions. *p*=0.023, Mann-Whitney *U* test. **I** Matrix Risk Score in young and old lungs in the absence of tumors, *p*=0.0020, two-sided Student’s *t* test

This analysis resulted in a 28-gene signature, with the microfibril protein microfibril-associated protein 4 (MFAP4) and the squamous-associated anchoring fibril collagen alpha1 type VII (COL7A1) having the lowest and highest odds ratios, respectively (Fig. 2A, Additional file 1: Fig. S2A and Table S1). In support of the feature selection approach, our signature includes ECM components identified as differentially regulated in non-small cell lung cancer, including osteopontin (SPP1), which has been associated with lung adenocarcinoma prognosis [79–83], but not yet specifically SqCC. Similarly, COL11A1 has also been associated with patient survival in NSCLC [84] and other cancers [85, 86]. Conversely, COL7A1 is known to play an important role in squamous tumorigenesis in the skin and esophagus [87, 88], suggesting that our signature captures SqCC-specific ECM remodeling programs.

Collapsing our matrix risk signature into a gene-weighted matrix risk score confirmed that this specific matrix risk score is significantly higher in tumor compared with non-tumor tissue in a test dataset partitioned from the TCGA LUSC dataset (Fig. 2B) as well as an independent SqCC dataset from the NCI-MD cohort (Fig. 2C) and distinguished tumor from non-tumor tissue by ROC analysis in these cohorts (Fig. 2D, E, Additional file 1: Fig. S2B). Analysis of each gene identified substantial predictive power for each individual gene within this risk signature, and further model refinement highlighted that a minimal signature of TNXB, COL7A1, and SPP1 achieved comparable predictive power in the NCI-MD cohort as measured by ROC curve analysis (Additional file 1: Fig. S2C), suggesting the key importance of these matrix components in tumor microenvironment ECM remodeling.

Comparatively, an adenocarcinoma-specific matrix signature developed on TCGA samples in parallel [44] shared 15 genes in common with the squamous matrix risk signature (Additional file 1: Table S1). This indicates that our matrix risk signature captures squamous-specific ECM components (e.g., COL7A1), as well as ECM components that associate with lung tumor development independently of histological subtype (e.g., EMILIN2, SPP1, COL10A1, COL11A1, and CTHRC1). To examine if our SqCC matrix risk signature reflected ECM changes in the lung specifically, or was more broadly applicable to other cancer types, we also examined its performance in distinguishing tumor from non-tumor tissue among solid tumor types in the TCGA Pan-Cancer cohort [45]. Our matrix risk score was significantly elevated in tumor compared with non-tumor tissue in all cancer types tested including lung adenocarcinoma (LUAD), except for pancreatic adenocarcinoma (PAAD) and sarcoma (SARC) (Additional file 1: Fig. S2D), and demonstrated predictive power (ROC analysis AUC>0.75) in multiple cancer types (Additional file 1: Fig. S2E and Table S2). Interestingly, the predictive value of our matrix risk signature was highest in the cervical SqCC and endocervical adenocarcinoma cohort, which has a large proportion of tumors with squamous characteristics [89]. This suggests that the matrisomal gene expression changes identified by our matrix risk signature may be more widely applicable to other cancer types, in particular those of squamous type, and warrants further investigation in these contexts.

To investigate which cell types express these key ECM genes, we calculated the enrichment of matrix risk genes with positive odds ratios (positive score) and negative odds ratios (negative score) in specific cell types identified by scRNAseq analysis of SqCC tumor and matched non-tumor tissue [46]. This analysis identified that core matrisomal genes that are positively associated with SqCC tumors (Additional file 1: Table S1; OR >1) are principally expressed by cancer-associated fibroblasts (CAFs) as well as cancer and myeloid cells (Fig. 2F, Additional file 1: Fig. S3A, B). In contrast, matrisomal genes that are negatively associated with SqCC tumors (Additional file 1: Table S1; OR <1) are expressed by alveolar and endothelial cells as well as a non-tumor-associated fibroblast population (Fig. 2G, Additional file 1: Fig. S3C). The contribution of these distinct fibroblast populations (cancer vs non-tumor-associated fibroblasts) to the ECM profile of SqCC tumors further supports the association of these matrix components with SqCC risk and may reflect phenotypic transdifferentiation of resident fibroblasts into CAFs during tumorigenesis [46]. Overall, these data suggest that changes in stromal cell gene expression within the tumor microenvironment, as well as matrisomal expression derived from cancer cells themselves, contribute to this risk score.

The early detection of pre-invasive SqCC in situ (CIS) lesions presents an opportunity to prevent the onset of lung cancer yet remains clinically challenging. While standard pathological assessment is able to distinguish between tumor and non-tumor tissue, there is currently no way of predicting which CIS lesions will progress to cancer, and which are likely to regress. This presents significant clinical challenges in deciding on the appropriate interventions while avoiding overtreatment [7]. Applying our matrix risk score to a recent dataset of pre-invasive CIS lesions [7] revealed that our matrix risk score was significantly higher in CIS lesions that progress to cancer compared to those that spontaneously regress (Fig. 2H). Further refinement of this matrix risk signature to specifically predict which lesions will progress to cancer identified a minimum 6-gene risk signature of RSPO1, CTHRC1, SPP1, MMRN1, COL10A1, and PRG4 as the most significant matrisomal predictors of premalignant progression with a ROC AUC of 0.99 (Additional file 1: Table S3, Fig. S3D). This suggests that not only are matrix changes occurring and detectable at the earliest pre-invasive stage of tumor development, but also that the ECM profile of CIS lesions has the potential to predict a high risk of tumor development.

Like many solid tumors, lung cancer incidence increases with age [78, 90, 91] and ECM of the lung continues to remodel with age [47, 92]. Age-related ECM remodeling is emerging as a significant contributor to the increased age-related cancer risk in other tumor types [93–95]. The matrix risk score was modestly although significantly correlated with age at diagnosis in lung cancer patients (Additional file 1: Fig. S3E). Further analysis of bulk RNAseq data from young and old lungs [47] revealed that older lungs have a higher SqCC matrix risk score than younger lungs (Fig. 2I). Therefore, matrix expression changes that occur during lung aging appear to overlap to some extent with matrix remodeling seen in lung tumors. In this way, age-associated ECM changes may prime aged lungs for SqCC initiation and progression, as was recently reported in melanoma [93–95], and thereby partially underpin the increased clinical incidence of these tumors with age.

Together, these data indicate that ECM remodeling occurs early in disease and is associated with malignant progression. Profiling these changes at the time of diagnosis may assist in identifying patients with high risk of developing aggressive lung cancer.

### SqCC matreotypes are prognostic

SqCC has a high rate of recurrence, even among early-stage patients following curative surgery. The ability to predict which patients are at a higher risk of recurrence would represent a significant milestone and has the potential to dramatically improve patient outcome. Expression-based molecular subtyping on the bulk tumor, encompassing cancer cells and other non-malignant cells within the tumor microenvironment, has revealed that SqCC tumors can be clustered into distinct tumor subtypes, with differences in biology and prognosis [24]. While these molecular subtypes broadly differ in cell adhesion and ECM pathways [24], recent studies indicate that they do not fully capture SqCC heterogeneity [27] and a detailed appraisal of the ECM landscape in SqCC may provide additional clinically actionable insight into SqCC biology. By focusing on the core matrisomal expression of SqCC tumors, we sought to define whether the matrix of the SqCC tumor microenvironment can be grouped into distinct matrix molecular subtypes (matreotypes), and to determine whether these matreotypes have prognostic value.

**Fig. 3:**
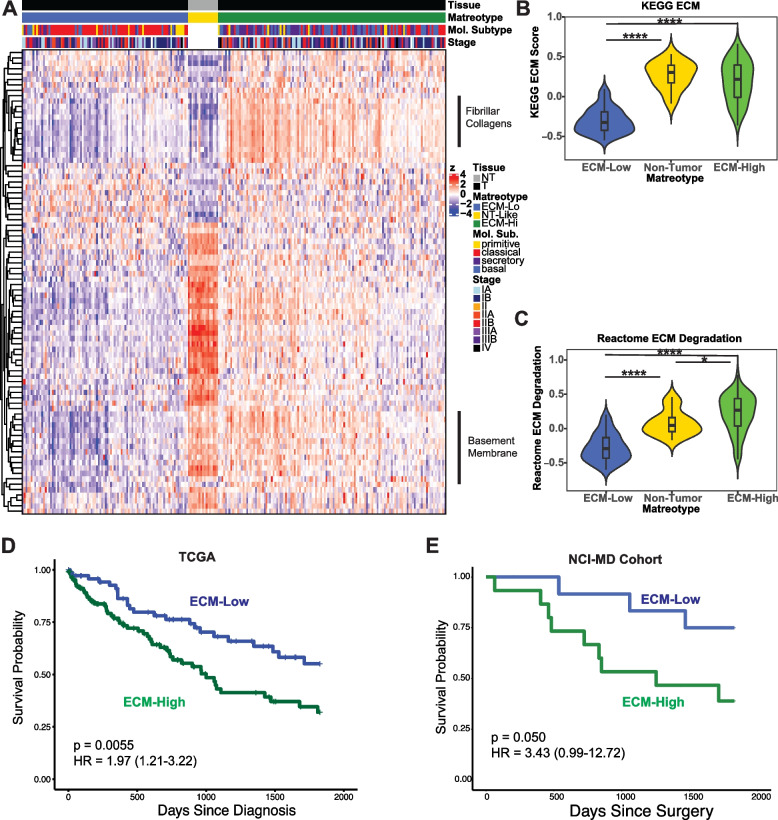
The ECM-High matreotype is associated with poor prognosis. **A** Gene expression heatmap of core matrisomal genes differentially expressed between tumor and non-tumor tissue illustrating the three major matreotypes (ECM-Low, Non-Tumor, and ECM-High) identified by consensus clustering. **B,C** ECM-specific signatures assigned to the three matreotypes reveal ECM-High and ECM-Low matreotypes compared with non-tumor (NT) matreotype for the KEGG ECM (**B**, ECM-Low vs ECM-High *p* <2.2E−16, NT-Like vs ECM-High *p*=0.27, ECM-Low vs NT-Like *p*=1.3E−10, Mann-Whitney *U* test) and Reactome ECM degradation pathways (**C**, ECM-Low vs ECM-High *p*<2.2E−16, NT-Like vs ECM-High *p*=0.011, ECM-Low vs NT-Like *p*=1.3E−7, Mann-Whitney *U* test). **D,E** Disease-specific survival of tumors corresponding to the ECM-High (blue) and ECM-Low (green) matreotypes in the TCGA (**D**, ECM-Low *n*=73; ECM-High *n*=107; Log-rank *p*=0.0055, Cox proportional hazards model HR (95% confidence interval)= 1.97 (1.21–3.22) of ECM-High compared with reference ECM-Low; multivariate model with age and stage covariates HR = 1.92 (1.17–3.17) *p*=0.010) and NCI-MD cohorts (**E**, ECM-Low *n*=14, ECM-High *n*=16; Log-rank *p*=0.050, Cox proportional hazards model HR (95% confidence interval)= 3.43 (0.99–12.72); multivariate model with stage covariate HR = 1.47 (1.01–18.55) *p*=0.048)

Consensus clustering of matrisomal genes was performed to identify tumor and non-tumor tissue with similar ECM gene expression profiles. This approach robustly revealed three clusters, with one cluster corresponding exclusively to non-tumor tissue (Fig. 3A, Additional file 1: Fig. S4A, B and C).

Broad inspection of the two tumor matreotypes identified one as enriched in matrisomal gene expression (designated ECM-High, Additional file 1: Table S4) and one depleted of matrisomal gene expression (designated ECM-Low, Additional file 1: Table S4), as reflected in the significant enrichment and depletion of both the KEGG ECM (Fig. 3B) and Reactome ECM degradation pathways (Fig. 3C) in the ECM-High and ECM-Low groups, respectively. Differential gene expression analysis identified that pro-fibrotic ECM genes are the most highly upregulated marker genes for the ECM-High matreotype (including COL5A1, COL1A2, THBS2, Additional file 1: Fig. S4D), while the ECM-Low matreotype is enriched in a small number of glycoproteins (including FNDC6 and BGLAP, Additional file 1: Fig. S4D). Importantly, upregulation of some matrix risk signature genes, including COL11A1, CTHRC1, COL10A1, and CILP2, in the ECM-High matreotype suggests some overlap between ECM remodeling profiles associated with risk and progression in SqCC.

Analysis of patient outcome for these two tumor matreotypes also identified distinct prognostic associations, with the ECM-high matreotype having significantly worse disease-specific survival than the ECM-low matreotype (Fig. 3D). These matreotypes were not significantly associated with tumor stage (Supplementary Table 4), with the poor survival of the ECM-High matreotype retained in the early-stage setting (Supplementary Figure 4E and F) suggesting that differentially prognostic matreotypes are independent of stage. Similarly, matreotypes were not significantly associated with clinical covariates gender, race, or smoking packyears (Additional file 1: Table S4). However, while there was a statistically significant difference in smoking behavior between patients with ECM-High and ECM-Low tumors, the predominance of smokers in SqCC cohorts (Additional file 1: Table S4) precludes robust assessment of matreotype associations with smoking behavior. Matreotype associations with smoking behavior will require future validation in larger cohorts with a greater diversity of smoking behavior, particularly a higher proportion of never smokers.

The prognostic association of SqCC matreotypes were independently validated in the NCI-MD cohort of largely early-stage SqCC (Fig. 3E, Additional file 1: Table S5) and a similar trend was also seen in the UHN cohort of early-stage tumors [20] (Additional file 1: Fig. 4G). This prognostic association was validated at the protein level in picrosirius-red-stained tissue microarrays of SqCC tumors imaged by polarized microscopy (Supplementary Figure 4H). When bound to fibrillar collagens (collagen types I, II, III and V), picrosirius red enhances collagen’s birefringent properties, enabling specific imaging of fibrillar collagens [96]. This enables selective imaging of fibrillar collagens, which include five of the top ten upregulated core matrisomal genes in the ECM-High compared with the ECM-Low matreotype (Additional File 1: Fig. 4H). Importantly, across all stages and in the early-stage setting of stage I and IIA tumors, multivariate Cox proportional hazards modeling indicates that a high picrosirius red birefringence signal is significantly associated with survival independently of stage (Additional file 1: Fig. S4I and J, Tables S6 and S7). Together, these data indicate that the ECM-high matreotype is significantly associated with poor overall survival in SqCC. Of note, analysis of the mutational landscape of these tumors did not identify any significant association of either matreotype with oncogenic driver mutations or FGFR amplifications (Additional file 1: Fig. S5A and B, Table S4). Thus, the matreotypes and their prognostic associations appear to be independent of the mutational landscape of the tumor.

**Fig. 4:**
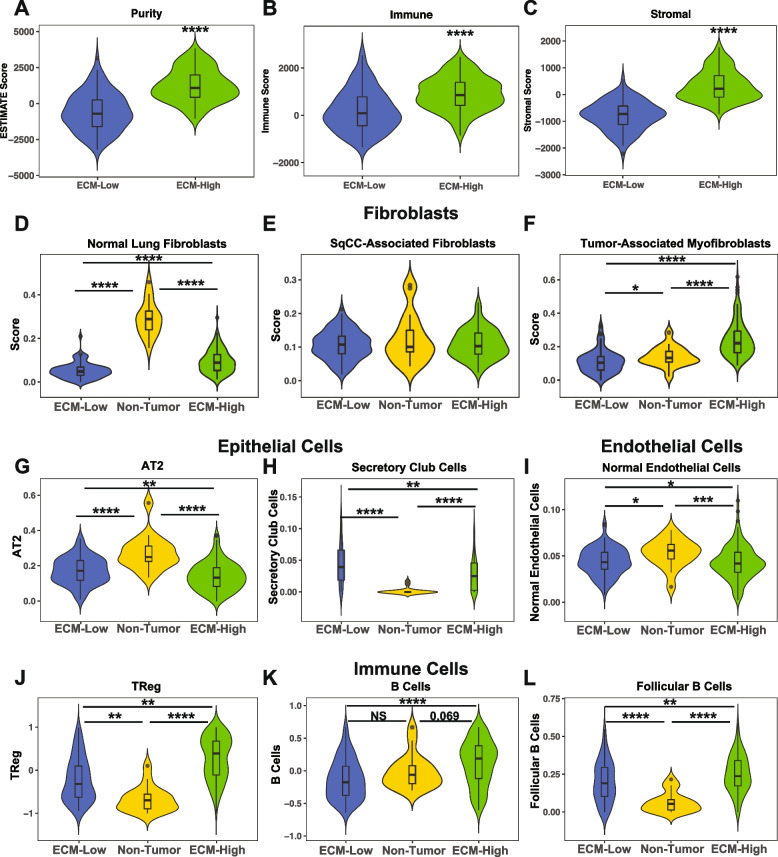
SqCC Matreotypes have distinct cellular ecosystems. **A–C** Comparison of tumor purity (**A**, *p*<2.2E−16, Mann-Whitney *U* test), immunological enrichment (**B**, *p*=8.0E−10, Mann-Whitney *U* test), and stromal enrichment (**C**, *p*<2.2E−16, Mann-Whitney *U* test) in the ECM-High (green) compared with ECM-Low (blue) matreotypes identifies significant enrichment of both stromal and immune cells in ECM-High matreotype tumors. **D–F** Fibroblast cell types in the three major SqCC matreotypes ECM-Low (blue), ECM-High (green), and non-tumor (yellow) with fibroblasts from non-tumor tissue (**D**, ECM-Low vs ECM-High *p*=3.8E−10, ECM-Low vs Non-tumor *p*=7.3E−11, ECM-High vs Non-Tumor *p*=7.8E−11, Mann-Whitney *U* test), SqCC-associated fibroblasts (**E**, ECM-Low vs ECM-High *p*=0.85, ECM-Low vs Non-tumor *p*=0.59, ECM-High vs Non-Tumor *p*=0.66, Mann-Whitney *U* test) and tumor-associated myofibroblasts (**F**, ECM-Low vs ECM-High *p*=2.3E−22, ECM-Low vs Non-tumor *p*=0.036, ECM-High vs Non-Tumor *p*=1.8E−5, Mann-Whitney *U* test). **G,H** Epithelial cell types in the three major SqCC matreotypes ECM-Low (blue), ECM-High (green), and non-tumor (yellow) corresponding to type II pneumocytes (**G**, AT2, ECM-Low vs ECM-High *p*=0.0018, ECM-High vs non-Tumor *p*=3.1E−7, ECM-Low vs Non-tumor *p*=6.6E−5, Mann-Whitney *U* test) and secretory club cells (**H**, ECM-Low vs ECM-High *p*=0.00042, ECM-High vs non-Tumor *p*=1.8E−6, ECM-Low vs Non-tumor *p*=2.5E−8, Mann-Whitney *U* test). **I** The ECM-High matreotype is significantly depleted of normal endothelial cells (ECM-Low vs ECM-High *p*=0.034, ECM-High vs non-Tumor *p*=0.00038, ECM-Low vs Non-tumor *p*=0.013, Mann-Whitney *U* test). **J–L** Immunological cell types in the three major SqCC matreotypes ECM-Low (blue), ECM-High (green), and non-tumor (yellow) corresponding to regulatory T cells (**J**, ECM-Low vs ECM-High *p*=9.7E−10, ECM-High vs non-Tumor *p*=1.5E−7, ECM-Low vs Non-tumor *p*=0.0060, Mann-Whitney *U* test), B cells (**K**, ECM-Low vs ECM-High *p*=4.9E−6, ECM-High vs non-Tumor *p*=0.069, ECM-Low vs Non-tumor *p*=1, Mann-Whitney *U* test), and particularly the follicular subset of B cells (**L**, ECM-Low vs ECM-High *p*=0.0044, ECM-High vs non-Tumor *p*=3.7E−8, ECM-Low vs Non-tumor *p*=5.5E−5, Mann-Whitney *U* test).

Analysis of canonical SqCC subtypes defined by Wilkerson et al. [24], namely primitive, classical, secretory, and basal subtypes, indicated that the ECM-Low matreotype is composed primarily of classical and primitive molecular subtypes, while the ECM-High matreotype was significantly enriched for secretory and basal subtypes (Table 2), consistent with cell adhesion and ECM receptor interactions as known features of basal SqCC [24]. These data suggest that SqCC matreotypes represent unique features of SqCC tumors, related to, but not entirely captured by, whole genome molecular subtyping.

**Table 2:** SqCC matreotype association with canonical SqCC molecular subtypes

Matreotype	Primitive	Classical	Secretory	Basal	***Predominant canonical subtype***
**ECM-Low**	12 (57.2%)	61 (64.9%)	7 (14.6%)	14 (23.2%)	Classical
**ECM-High**	9 (42.8%)	33 (35.1%)	41 (85.4%)	46 (76.7%)	Secretory/basal
***P-value***	0.167	5.18E−9	6.67E−9	6.93E−4	

As pro-fibrotic ECM remodeling identified in the ECM-High matreotype has been associated with worse prognosis in a number of different cancer types, including pancreatic and breast cancer [97], we sought to examine the prognostic value of these matreotypes in other solid tumor types, including lung adenocarcinoma. This pan-cancer analysis revealed that our matreotypes are not prognostic in lung adenocarcinoma (Additional file 1: Figure S5C), attesting to the subtype specificity of these matreotypes in NSCLC. However, the ECM-High matreotype was significantly associated with disease-specific and progression-free survival in a number of other solid tumor types, including pancreatic ductal adenocarcinoma (PAAD) (Additional file 1: Fig. S5D and E, Table S8). This suggests that ECM remodeling associated with these matreotypes may play an important role in the progression of a specific but diverse subset of solid tumors.

### Prognostic SqCC Matreotypes have divergent cellular ecosystems

Canonical molecular subtypes are thought to reflect different cellular compositions [24, 25] and since these matreotypes were identified in bulk RNAseq data, gene expression values reflect the expression of multiple cell types within the tumor microenvironment. To determine how different cell types within the tumor microenvironment may be contributing to the different ECM compositions of these matreotypes, we utilized cellular deconvolution approaches to infer the cellular composition of these tumors.

In silico estimation of tumor purity using ESTIMATE [61] revealed significantly lower tumor purity in the ECM-High matreotype compared with the ECM-Low matreotype (Fig. 4A). This lower tumor purity was due to significantly higher stromal (Fig. 4B) and immune scores (Fig. 4C) in the ECM-High compared with the ECM-Low matreotype, suggesting that ECM-High matreotype tumors may harbor a more diverse cellular ecosystem. Of note, tumor purity itself (*p*=0.2, HR = 1 [0.99–1], Cox proportional hazards model) and immune scores (*p*=0.72, HR = 1 [0.99–1]) were not significantly associated with survival, and the stromal score (*p*=0.039, HR = 1.0003 [1–1.0006]) was only marginally associated with survival. This indicates that broad enrichment of stromal cells within the tumor microenvironment does not explain the prognostic value of the matreotypes and highlights that the specific matrisomal profile of SqCC may be a stronger indicator of prognosis than tumor purity alone.

To further interrogate these differences in more detail, deconvolution approaches were applied using cell type-specific signatures from NSCLC scRNAseq data [46] using CIBERSORTx [98]. While several fibroblast subtypes (subtypes 2 (COX4I2-high), 4 (PLA2GA-high) and 5 (MMP3-high) as identified in [46]) were not detected in more than 10% of samples in this cohort, normal fibroblasts were significantly depleted in both tumor matreotypes compared with the non-tumor matreotype (Fig. 4D). Furthermore, the ECM-High matreotype was significantly enriched with tumor-associated myofibroblast cells (Fig. 4F) but not a fibroblast subset originally identified in a subset of SqCC tumors but currently with no known functional significance (Fig. 4E). Additionally, the ECM-High matreotype was significantly depleted of type II pneumocytes (AT2, Fig. 4G), and secretory club cells (Fig. 4H) relative to the ECM-Low matreotype, suggesting the presence of tumor-associated epithelial dysregulation in these poor-prognosis tumors. As expected, the ECM-High matreotype was significantly depleted of normal endothelial cells (Fig. 4I), consistent with high expression of correlation cluster 2 genes (Fig. 1D) in this subset of tumors.

Direct comparison of immune cell scores in the ECM-High and ECM-Low matreotypes using NSCLC-specific immune signatures [51] indicated that the ECM-High matreotype is relatively enriched in a variety of immune cells compared with the ECM-Low matreotype, but levels remain lower than non-tumor tissue (Additional file 1: Fig. S6). However, of particular note was that this matreotype was significantly enriched with regulatory T cells (Fig. 4J), B cells (Fig. 4K), and the follicular B cell subset (Fig. 4L) compared with both non-tumor tissue and the ECM-Low matreotype. Interestingly, T-follicular helper cells (Additional file 1: Fig. S6A) were also significantly enriched in the ECM-High matreotype, suggesting the potential enrichment of tertiary lymphoid structures in this matreotype. Furthermore, a higher MHC Class II expression score in the ECM-High matreotype (Additional file 1: Fig. S6E) suggests the potential for differences in immunological activation in these matreotypes. While regulators of antitumoral immune surveillance are emerging, including matrix-mediated T cell exclusion [99], these data warrant further functional and clinical investigations into the potential for matreotypes to inform the prioritization of patients for immune checkpoint therapies.

Together, these data suggest that cellular heterogeneity in SqCC may contribute to differences in the ECM profile of the tumors. The differences in these cellular ecosystems have potential implications for the tumor etiology and the efficacy of therapeutic interventions, and in particular immune checkpoint inhibitors.

### Matrisomal features are associated with pro-invasive signaling

**Fig. 5:**
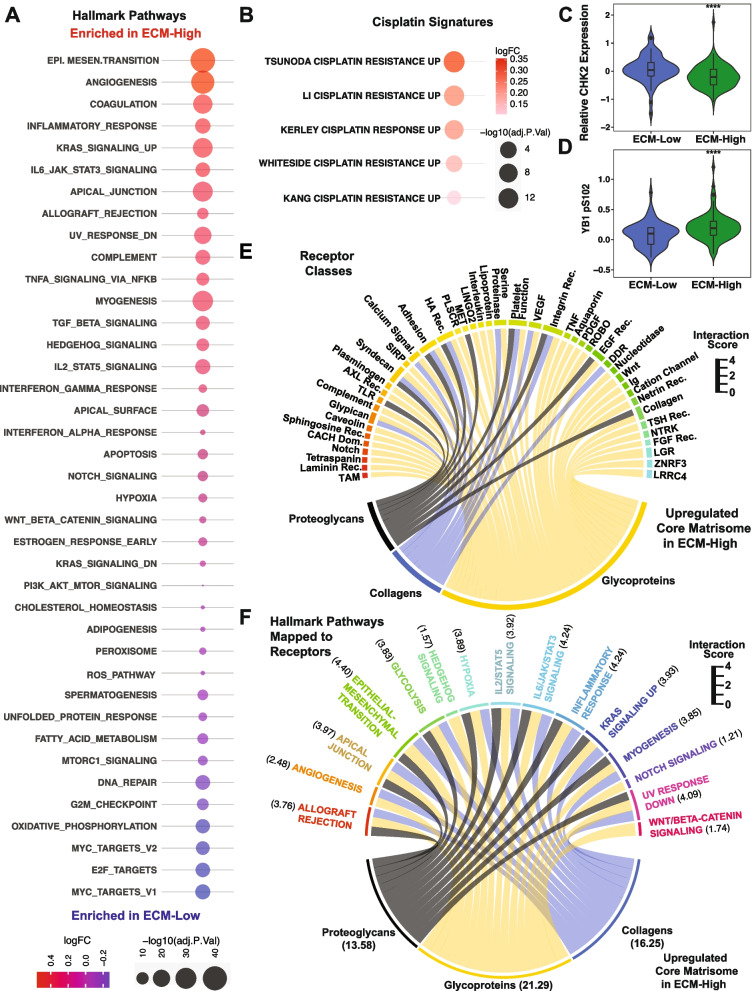
ECM components contribute to signaling pathways associated with prognosis. **A** Top 25 up- and downregulated differentially enriched MSigDb hallmark pathways in the ECM-High matreotype compared with the ECM-Low matreotype. Circle size indicates the inverse of the −log10(*p*-value), color reflects the log fold change (logFC) of the ECM-High matreotype compared with the ECM-Low matreotype. **B** Pathway analysis of cisplatin-related signatures comparing ECM-High matreotype with the ECM-Low matreotype. **C** Abundance of CHK2 in the ECM-High matreotype (green) compared with the ECM-Low matreotype (blue). *p*=1.5E−4, Mann-Whitney *U* test. **D** Comparison of the phosphorylated YB1(S102) in the ECM-High matreotype (green) compared with the ECM-Low matreotype (blue). *p*=1.1E−3, Mann-Whitney *U* test. **E** Ligand-receptor interaction results of differentially expressed core matrisomal genes significantly upregulated in ECM-High compared with ECM-Low matreotypes and the receptor groups that they directly interact with. Sector width is the aggregated log fold change of the ligand-receptor interaction strength comparing the ECM-High to ECM-Low matreotype. The scale indicates the width of each link between the ECM category and its cognate receptors. The widths of each link are the maximum fold change of the ligand-receptor interaction scores comparing the ECM-High and ECM-Low groups for that ligand-receptor interaction. **F** Hallmark pathways regulated by ligand-receptor interactions that are significantly enriched in the ECM-High compared with the ECM-Low matreotypes. The scale indicates the width of each link between the ECM category and the hallmark pathway. The widths of each link are the maximum fold change of the ligand-receptor interaction scores comparing the ECM-High and ECM-Low groups for that hallmark pathway

To further examine the distinct tumor biology associated with these matreotypes, we performed pathway enrichment analysis comparing the ECM-Low and ECM-High groups using the MSigDb Hallmark pathway database. As expected, ECM-related pathways were the most highly enriched in the ECM-High matreotype (Additional file 2: Table S1). Examination of non-ECM-associated pathways revealed multiple differentially expressed hallmark pathways, including enrichment of inflammatory, epithelial-to-mesenchymal transition, angiogenesis, and cell-cell adhesion pathways in the ECM-High matreotype (Fig. 5A). Comparatively, the ECM-Low matreotype was enriched in DNA repair pathways, including the G2/M checkpoint as well as ROS signaling (Fig. 5A).

DNA damage pathways play a significant role in regulating resistance to conventional chemotherapy agents, including platinum-based agents which are a frontline therapy for SqCC [100]. Pathway analysis of ECM-High and ECM-Low matreotypes using the MSigDb C2 oncogenic database (Additional file 1: Fig. S7A, Additional file 2: Table S2), which includes signatures specific to tumor biology and therapeutic responses, identified significant enrichment of multiple cisplatin resistance gene signatures in the ECM-High matreotype compared with the ECM-Low matreotype (Fig. 5B), suggesting that DNA damage checkpoint deficiencies in ECM-High tumors may contribute to poor treatment response in these patients. Analysis of reverse phase protein array proteomic TCGA data in these same tumors [42] indicated significantly reduced expression of the double-stranded break response protein CHK2 (Fig. 5C) and increased activation of YB1 (pS102, Fig. 5D) in ECM-High matreotype tumors compared with ECM-Low matreotype tumors at the protein level. CHK2 and YB1 are known mediators of platinum resistance [101–103] and may contribute to poor platinum response in ECM-High tumors.

ECM components directly interact with cell surface receptors to regulate the activity of numerous signaling pathways, including those involved in epithelial-to-mesenchymal transition and ECM production [35]. Ligand-receptor interaction analysis was performed to gain insight into the potential direct effects of these matreotypes on outside-in signaling. A database of curated ligand-receptor interactions [65] was mined for interactions between core matrisomal genes differentially expressed between ECM-High and ECM-Low SqCC tumors and their cognate receptors. An interaction strength was assigned to each core matrisomal ligand-receptor interaction based on their expression levels, to infer the potential for core matrisome-driven receptor activation. Receptors were then grouped into functional classes and mapped to signaling pathways by their MSigDb hallmark signatures. The ECM profile of the ECM-High matreotype was associated with significant enrichment of multiple receptor classes (Fig. 5E, Additional file 2: Table S3) involved in diverse signaling pathways. The most significantly enriched of these core matrisome-receptor pairs were fibrillar collagens (COL1A2, COL1A1, COL5A1, COL5A2) interacting with receptors FLT4/VEGFR3, integrins α1, α11, and β1 as well as CD93 (Additional file 2: Table S3). Conversely, only interactions between the glycoprotein ZP3 and EGF and MERTK (TAM family) receptors were identified in the ECM-Low matreotype (Additional file 1: Fig. S7B), although the strength of these interactions was not significantly higher compared with the ECM-High matreotype. Importantly, ligand-receptor interactions that were significantly enriched in the ECM-High matreotype (Fig. 5E) could be mapped to significantly upregulated hallmark pathways in this matreotype (Fig. 5F) (including epithelial to mesenchymal transition, apical junction, and myogenesis (Fig. 5A)) suggesting that core matrisome components have the potential to contribute to the upregulation of these biological pathways within different SqCC matreotypes. Together, these data indicate the potential for the ECM profile of different SqCC to regulate cancer cell biology and the mechanisms that promote tumor progression.

### The pro-fibrotic matrisome is associated with pro-invasive signaling in poor-prognosis SqCC

Integrins represented the largest class of receptors with the potential to be activated by the various matrix elements of the ECM-High matreotype. Integrins have been demonstrated to play a role in NSCLC tumorigenicity [104, 105] as well as activating epithelial to mesenchymal signaling to support cancer cell metastasis and in activating fibroblasts to a myofibroblast-like state seen in cancer-associated fibroblasts.

**Fig. 6:**
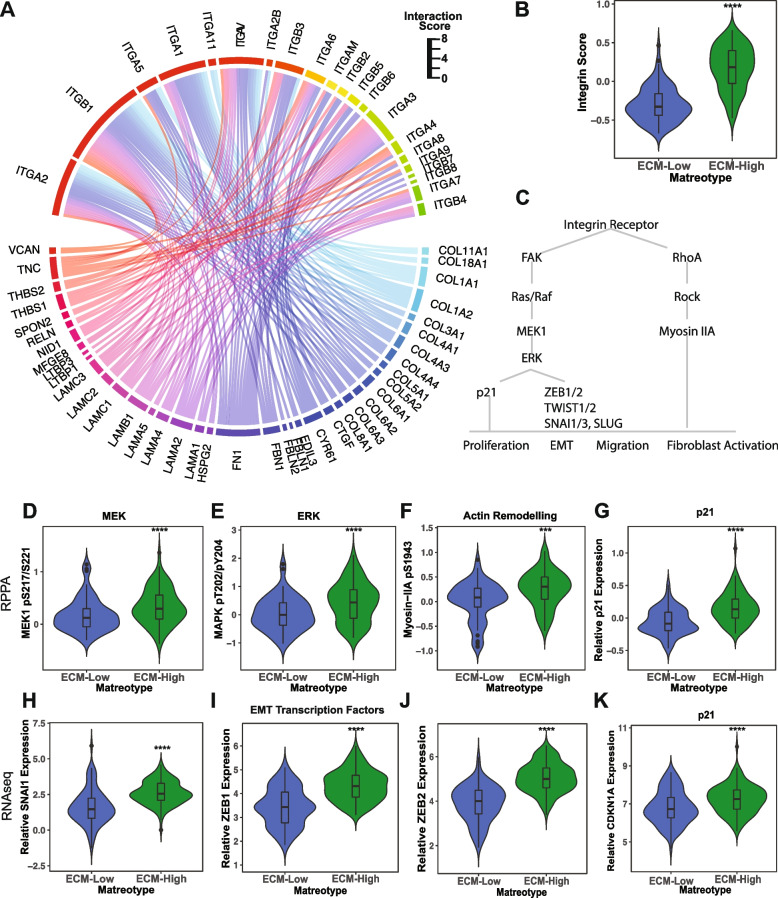
ECM-driven integrin signaling is associated with EMT and fibroblast activation in the ECM-High matreotype. **A** Ligand-receptor interaction results of differentially expressed core matrisomal genes and their cognate integrin receptors upregulated in ECM-High compared with ECM-Low matreotypes. Sector width is the aggregated log fold change of the ligand-receptor interaction strength comparing the ECM-High to ECM-Low matreotype. **B** Integrins are upregulated in the ECM-High compared with the ECM-Low matreotypes as indicated by the integrin receptor score. *p*=1.3E−23, Mann-Whitney *U* test. **C** Schematic of integrin-mediated signaling pathways active in the ECM-High matreotype. **D–G** Comparison of integrin-dependent signaling in the ECM-High (green) and ECM-Low (blue) matreotypes as indicated by the RPPA abundance of MEK1 pS217/S221 (**D**, *p*=1.6E−3), ERK pT202/204 (**E**, MAPK pT202/204, *p*=3.4E−4), Myosin IIA pS1943 (**F**, *p*=0.00027), and p21 (**G**, *p*=8.1E−8), Mann-Whitney *U* test. **H–K** Expression of genes SNAI1 (**H**, *p*=7.3E−12), ZEB1 (**I**, *p*=9.9E−15), ZEB2 (**J**, *p*=1.9E−21), and P21/CDKN1A (**K**, *p*=0.00014) in RNAseq data in the ECM-High (green) and ECM-Low (blue) matreotypes; Mann-Whitney *U* test. *** *p*<0.001, ****p<0.0001

Collagens, proteoglycans, and glycoproteins that were enriched in the ECM-High matreotype were identified as ligands for a range of integrin receptors (Fig. 6A), with known functional roles in cell survival, migration, and invasion required for metastatic relapse and in fibroblast activation [106]. Similarly, integrin receptors themselves (Fig. 6B), as well as components of the integrin adhesome (Additional file 1: Fig. S8A), were also overexpressed in the ECM-High matreotype compared with the ECM-Low matreotype (Fig. 6B, C), suggesting the potential for further reinforcement of integrin-mediated signaling in these tumors.

In accordance with elevated integrin signaling in this matreotype, interrogation of TCGA RPPA proteomic data from these tumors identified significant phosphorylation of integrin phospho-adhesome component PKCdelta [107] (pS664, Additional file 1: Fig. S6B) as well as activation of the MEK/ERK signaling pathway through increased activation of MEK1 (pS217/221) and ERK1/2 (p202/204, Fig. 6D–G). Similarly, we observed increased expression of MEK/ERK downstream targets p21 (protein: Fig. 6G; transcript: Fig. 6K)) and master EMT regulators of the ZEB (ZEB1, ZEB2), SNAIL (SNAI1, SNAI2), and TWIST (TWIST1, TWIST2, and SLUG (SNAI3)) transcription factor families (Fig. 6H–J and Additional file 1: Fig. S8C). It should be noted that other receptors, such as EGFR, can also activate MEK/ERK signaling and may also contribute to these proteomic phenotypes. Similarly, since these downstream effectors and transcription factors are also involved in integrin-dependent and integrin-independent fibroblast activation, higher levels of these factors may also reflect increased activated fibroblasts within ECM-High tumors. Interestingly, high expression of integrin/MEK/ERK target p21 has also been implicated in mediating cisplatin resistance in NSCLC and other cancers [108, 109] suggesting that elevated integrin signaling as a result of an ECM-High matreotype could underpin the chemotherapy resistance predicted for these tumors (Fig. 5B).

In addition, myosin IIA phosphorylation was significantly upregulated in the ECM-High matreotype (Fig. 6F). Myosin IIA activation supports cancer cell migration to promote metastasis in NSCLC [110, 111] and is also associated with cancer-associated fibroblast activation [112] warranting further mechanistic validation of these signaling events in supporting metastatic propensity and fibroblast activation in the ECM-High matreotype.

### The matrisome contributes to fibrogenic signaling in SqCC

In addition to impacting cancer cell behavior, elevated integrin activation driven by matrisomal components of the ECM-High matreotype (Fig. 6) may also promote fibroblast to myofibroblast transdifferentiation [113–115] that is thought to contribute to the expression of pro-fibrotic ECM components in the ECM-High SqCC. In this way, integrin signaling amplified by the core matrisome may perpetuate increased pro-fibrotic ECM remodeling and subsequent pro-metastatic signaling.

**Fig. 7:**
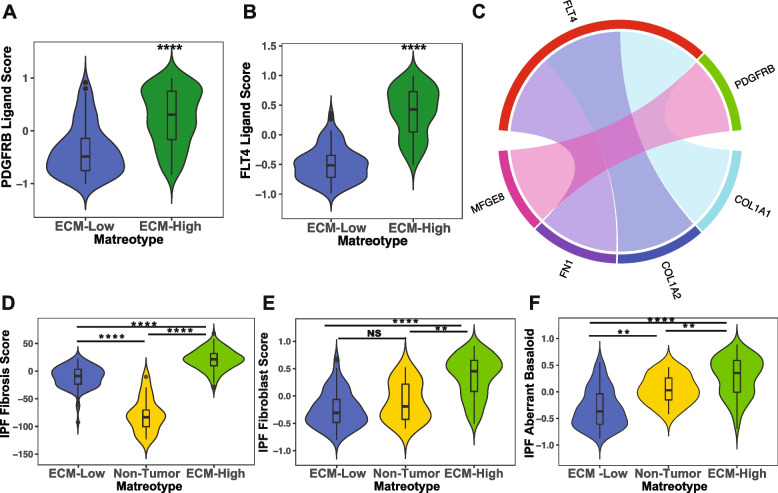
The poor-prognosis matreotype overlaps with ECM remodeling in idiopathic pulmonary fibrosis. **A,B** ECM genes that act as ligands for the PDGFRB (**A**, *p*=3.7E−15) and FLT4 (**B**, *p*=8.3E−31) receptors in the ECM-High and ECM-Low matreotypes; Mann-Whitney *U* test. **C** Ligand-receptor interactions between core matrisome genes differentially expressed between ECM-High and ECM-Low matreotypes and the fibrogenic receptors FLT4 and PDGFRB. **D** IPF fibrosis score in the ECM-Low (blue), ECM-High (green), and non-tumor (yellow) matreotypes; ECM-High vs ECM-Low *p*<2.2E−16, ECM-High vs Non-tumor *p*=3.3E−11, ECM-Low vs Non-tumor *p*=2.8E−9, Mann-Whitney *U* test. **E** Estimated abundance of IPF-specific CTHRC1+ fibroblasts in the ECM-Low (blue), ECM-High (green), and non-tumor (yellow) matreotypes; ECM-High vs ECM-Low *p*=4.1E−20, ECM-High vs Non-tumor *p*=0.0011, ECM-Low vs Non-tumor *p*=1.0, Mann-Whitney *U* test. **F** IPF Aberrant basaloid score in the ECM-Low (blue), ECM-High (green), and non-tumor (yellow) matreotypes (ECM-High vs ECM-Low *p*=8.9E−19, ECM-High vs Non-tumor *p*=0.0062, ECM-Low vs Non-tumor *p*=0.0056), Mann-Whitney *U* test

The TGFb, PDGF, FGF, and VEGF signaling pathways are also well characterized fibrogenic pathways that can induce the transdifferentiation of fibroblasts towards a myofibroblast-like phenotype observed in NSCLC fibroblast subsets [116–118]. In order to understand the potential direct impact of these matreotypes on pro-fibrogenic receptors in the tumor microenvironment, we performed a focused analysis of the interactions between core matrisomal genes that are significantly enriched in the ECM-High matreotype and the fibrogenic PDGF, FGF, and VEGF receptors. This analysis revealed that collagen I, fibronectin, and the milk fat globule EGF and factor V/VIII domain containing glycoprotein MFGE8, which are highly expressed in ECM-High tumors, interact with the PDGFRB and FLT4 receptors (Fig. 7A, B). High PDGFR pathway activity in the ECM-High matreotype is also reflected in significant enrichment of signatures for response to PDGFR inhibition by imatinib compared with the ECM-Low matreotype (Additional file 1: Fig. S9A, Additional file 2: Table S2).

Therefore, in addition to integrin- and biomechanical-induced fibrogenic signaling, direct interaction of the core matrisome with PDGF and VEGF receptors may also activate fibrogenic signaling thereby amplifying the deposition of pro-fibrotic ECM associated with poor prognosis in SqCC patients.

### Prognostic matreotype overlap with idiopathic pulmonary fibrosis

Fibrogenic signaling through these tyrosine kinase receptors is known to play a role in the progression of idiopathic pulmonary fibrosis [119], a chronic, degenerative lung condition characterized by extensive ECM remodeling that typically presents at older age and is associated with increased risk of developing lung cancer [120].

Although the precise molecular drivers of IPF remain unclear, parallels have been drawn between the pathogenesis of IPF and lung cancer and ECM remodeling has been implicated in increased lung cancer risk in these patients [121]. We sought to understand to what extent the matrisomal profile of the poor-prognosis ECM-High matreotype overlaps with that of IPF. We applied an IPF-derived fibrosis score developed from human IPF lung transcriptomic data [48] to SqCC. Our analysis showed that tumor tissue had a higher fibrosis score than non-tumor tissue and that this fibrosis score was highest in the ECM-High matreotype (Fig. 7D). This indicates that the ECM remodeling associated with poor prognosis in NSCLC overlaps to some extent with ECM remodeling that defines IPF.

Phenotypic reprogramming of fibroblasts towards a pro-fibrogenic state, with enhanced proliferative and invasive capacity, is believed to drive progressive lung fibrosis in IPF [122]. These IPF-specific fibroblasts have phenotypes that are distinct from normal lung fibroblasts and are characterized by high expression of fibrotic ECM genes such as COL1A1 and CTHRC1 [49, 123]. Deconvolution of bulk RNAseq data in tumors identified that the ECM-High matreotype was significantly enriched with the transcriptomic profile of the CTHRC1+ IPF-specific fibroblast population (Fig. 7E) thought to be important in IPF pathogenesis [49]. Similarly, the ECM-High matreotype was significantly enriched in IPF-specific aberrant basaloid cells identified exclusively in IPF lungs (Fig. 7F), which are thought to result from epithelial dysregulation in IPF and contribute to aberrant fibroblast phenotypes in this disease [50]. Together, this suggests that fibroblast and epithelial dysregulation featured in IPF pathogenesis may also operate in a subset of poor-prognosis SqCC, contributing to matrisomal dysregulation and tumor progression.

Overall, our analyses have identified ECM profiles of CIS lesions and carcinomas that are associated with progression and poor prognosis. Overlap in the ECM components that are represented in the matrix risk signature and are upregulated in the ECM-High matreotype suggest that early ECM remodeling programs initiated prior to the onset of, or very early on, in malignancy may establish an ECM profile that supports aggressive tumor behavior and that persists throughout tumor progression. In silico analysis raises the notion that an amplification loop of fibrotic ECM production, integrin activation, and pro-fibrogenic processes has the potential to promote chemotherapy resistance and metastatic recurrence in a subset of poor-prognosis SqCC. An improved understanding of the ECM landscape in SqCC may identify more effective therapeutic strategies that co-target the tumor-supporting components of the ECM.

## Discussion

Treatment options and efficacy remain limited in the SqCC subtype. The lack of targetable mutations in these tumors has hindered progress in the development of more effective, personalized treatment approaches that have revolutionized patient outcome in subsets of lung adenocarcinoma patients [4]. Furthermore, significant tumor heterogeneity [9] and the high mutational burden of these tumors [10] make it challenging to achieve sustained clinical responses by targeting tumor cells alone [11, 12]. Understanding how the tumor microenvironment contributes to tumor progression may reveal opportunities to target the tumor-supporting elements of this microenvironment in order to improve treatment efficacy. Furthermore, it is likely to assist in the identification of patients at higher risk of developing aggressive disease, or of relapsing. Through focused characterization of the tumor ECM, our analysis has revealed that subtype-specific ECM remodeling is associated with tumor initiation and progression in SqCC, uncovering potential opportunities to improve the diagnosis and treatment of subsets of SqCC patients with the poorest prognosis.

Components of the ECM form a highly interconnected and dynamic arrangement of macromolecules [35]. It is increasingly appreciated that the architecture of these components and their association with one another, not just their abundance, can induce heterogeneous effects on cell behavior [31]. Our matrix risk signature has defined key ECM genes that distinguish between tumor and non-tumor tissue in SqCC, as well as in lung adenocarcinoma and various cancer types. Our analysis indicates that these key ECM components are highly correlated with many other ECM components, and together with their known scaffolding functions with other ECM molecules, suggests that tumor- and aging-associated changes in the expression of these key matrix components likely result in profound changes in the organization of the lung ECM. These cumulative changes then feed into SqCC progression. We identified significant overlap in the SqCC and adenocarcinoma risk signatures for matrix components that regulate collagen fibril architecture including COL10A1, COL11A1, and CTHRC1 [124] suggesting that our risk signature represents changes in the organization of fibrillar collagens. COL10A1, COL11A1, and CTHRC1 were also identified in a matrix-specific NSCLC subtype-independent prognostic signature [17], supporting the involvement of these matrix components in lung tumorigenesis generally. EMILIN2, which is thought to confer elasticity to tissues and modulate receptor signaling, was identified as downregulated in both SqCC and adenocarcinomas. While its function in non-small cell lung tumors is not currently known, EMILIN2 has been associated with poor prognosis in gastric cancer where it modulates cancer cell apoptosis and tumor angiogenesis [125]. The glycoprotein osteopontin SPP1 was also identified as differentially expressed in both squamous and adenocarcinoma tumors. SPP1 has recently been identified as a marker of macrophage polarization and implicated in mediating immune evasion [79] and immunotherapy response [126] consistent with immunological mechanisms regulating lung tumorigenesis [8]. However, our analysis also identified core matrisome components that were associated with SqCC tumors but not adenocarcinoma tumors. These squamous-specific components include COL7A1, which is known to play an important role in squamous tumorigenesis in the skin and esophagus [87, 88], and COL4A6, which we identified as correlating with the expression of squamous master regulator TP63. While an integrated picture of how these matrix components collectively regulate ECM composition and architecture in lung tumors generally, as well as in the specific SqCC and adenocarcinoma subtypes, is not yet clear, the applicability of this risk signature to non-lung primary tumors raises the notion that tumorigenesis may involve overlapping ECM remodeling processes across diverse tumor types.

The implementation of lung cancer screening has improved mortality through the early detection of overt tumors, as well as pre-invasive lesions, which are more prevalent in screening programs than positive tumor diagnoses [127, 128]. Pre-invasive lesions, which are dysplastic but are not yet invasive [129], spontaneously regress in approximately 30% of patients [7]. They are typically periodically monitored for accelerated growth and the acquisition of invasive characteristics before interventions are recommended. There are currently no markers for those premalignant lesions that are likely to progress to cancer, and as such the clinical management of these patients to avert the initiation of lung cancer remains challenging. Our analysis indicates that CIS lesions that progress to SqCC, which histologically are indistinguishable from those CIS lesions that will spontaneously regress, have acquired tumor-like ECM profiles. Identification of these features during monitoring may enable patients with high-risk premalignant lesions to be prioritized for surgical intervention to prevent the onset of invasive SqCC. Our analysis of fibrillar collagens by picrosirius red staining of tissue microarrays suggests that such an approach, which could integrate with existing pathological processing of tumor samples, could be used to prognosticate tumors by matreotype. It remains to be determined if key ECM markers from the matrix signatures could also be assessed at the protein level using immunohistochemistry for the prognostication of premalignant lesions (risk signature), and also at SqCC diagnosis (matreotyping as ECM-High or ECM-Low). Immunohistochemistry staining for specific ECM markers identified in our analysis may be more compatible with existing pathological analysis pipelines than transcriptomic approaches. This will be integral for rapid implementation of ECM profiling for the prioritization of high-risk patients for more rigorous intervention or stromal co-targeting therapies.

Growing evidence points to immune surveillance as a major regulator of premalignant progression in SqCC [8]. With this in mind, the glycoprotein SPP1, which we identified as positively associated with SqCC risk, is produced by tumor-associated macrophages in vitro and induces PD-L1 expression in lung adenocarcinoma [79]. This suggests that the immunomodulatory activity of ECM components in our matrix risk signature may contribute to immune evasion mechanisms that enable premalignant progression. As has been demonstrated in other tumor types, the arrangement of multiple ECM components such as fibrillar collagens and their associated matrix molecules has the potential to also regulate immunological surveillance. For example, fibrillar collagen architecture, which is regulated by several ECM components in our risk signature (including COL11A1, CTHRC1, and COL10A1), is also associated with altered T cell-mediated immune surveillance [95, 130, 131]. Preliminary data also suggests that a COL11A1-expressing CAF subset may also promote T cell exclusion from the tumor microenvironment [132]. How these ECM components influence anti-tumor immune surveillance will be an important consideration in understanding and predicting the immune checkpoint therapy response.

Changes in collagen architecture also occur during lung aging [47, 92]. The enrichment of our matrix risk signature in older compared with younger lungs supports the notion that age-related ECM remodeling may prime lung tissue for lung tumor initiation and underpin the increased incidence of lung cancer with age. Age-related changes in dermal collagen architecture induced by HAPLN1 has been linked to reduced T cell motility and infiltration, and increased melanoma incidence with age [93, 95], raising the possibility that ECM remodeling represented by our matrix risk signature captures a similar process in SqCC. Whether ECM components that constitute the risk signature reflect or regulate immune surveillance to enable unhindered progression of dysplastic lesions warrants further investigation.

Several of the matrix components identified in the matrix risk signature are also significantly overexpressed in our prognostic ECM-High matreotype that constitutes a subgroup of SqCC patients with the worst prognosis. This, together with the stage independence of these matreotypes, suggests that ECM remodeling during the initiation of lung tumors may establish an ECM remodeling program that supports subsequent aggressive tumor growth and metastasis that persists throughout tumor progression. Our identification of matrisomally driven integrin and fibrogenic signaling in this ECM-High matreotype suggests that the initiation of the pro-fibrotic ECM remodeling generates a positive feedback loop to promote further fibrotic ECM deposition and activation of these pro-metastatic signaling pathways.

The association of the ECM-enriched, ECM-High matreotype with worse prognosis is consistent with an observed association of increased peritumoral stroma (as defined by H&E pathology) with worse overall and recurrence-free survival, and significantly reduced expression of the EMT marker E-cadherin in SqCC [133]. Early establishment of these ECM remodeling programs is also consistent with observations that pro-fibrotic gene expression changes occur early in SqCC progression, coinciding with the acquisition of an invasive phenotype [134], and therefore may enable early cancer cell dissemination from the primary site. Furthermore, numerous studies support the association of pre-existing fibrotic ECM remodeling from chronic lung diseases with worse lung cancer survival [135, 136], potentially due to increased invasion in these tumors [135]. Our inference of matrisome-driven receptor activation suggests that the ECM itself has the potential to further amplify integrin signaling to support the metastatic spread of cancer cells and the activation of fibroblasts and warrants further functional studies to dissect the contribution of integrin signaling in these cell types to tumor biology.

Advanced stage SqCC and adenocarcinoma that lack targetable mutations and are candidates for immune checkpoint inhibitors are largely treated in a similar manner. However, our analysis indicates that prognostic ECM remodeling programs in SqCC are distinct from those in lung adenocarcinoma. While adenocarcinoma has been reported to be more fibrotic than SqCC [137], our analysis indicates that it is the upregulation of other specific core matrisomal components, not just fibrillar collagens, that are prognostic in SqCC. The distinct biology of these two NSCLC subtypes is supported by a recent proteogenomic study that identified the majority of SqCC clustered distinctly from adenocarcinoma subtypes [27]. That these tumors respond differently to the matrix environment may reflect the fact that SqCC and adenocarcinomas typically arise in different regions of the lung (and likely different cells of origin [138]), which are characterized by a different ECM composition [139]. Lung fibroblasts within these different compartments also display distinct phenotypes, and fibroblasts derived from SqCC tumors accumulate more than adenocarcinoma-associated fibroblasts via beta-1 integrin-dependent mechanisms [114], which may also accelerate matrisome-mediated pro-fibrogenic signaling in the ECM-High matreotype. Differential effects of the ECM on lung cancer histology have also been noted in the context of LKB1-negative tumors, where LOX-dependent collagen crosslinking can drive the transdifferentiation of adenocarcinomas to SqCC [140], although the precise mechanism by which the ECM potentially modulates lung cancer histology remains unclear.

Our poor-prognosis ECM-High matreotype has an ECM profile which overlaps with IPF and appears enriched for an IPF-specific fibroblast phenotype, suggesting that matrix remodeling in this subset of SqCC with the worst prognosis may be driven by similar mechanisms as those underlying IPF. Enrichment of the IPF-specific aberrant basaloid cell signature in the ECM-High matreotype may also reflect the known epithelial dysregulation associated with SqCC and the transformation of basal cells, which are the likely cells of origin in SqCC [138]. Interestingly, this epithelial cell type has high expression of EMT markers and p21 [50], which were identified as downstream of elevated integrin signaling in our ECM-High matreotype. The physical proximity of aberrant basaloid cells covering myofibroblastic foci in IPF lungs [50] raises the possibility that crosstalk between neighboring IPF-like fibroblasts and transformed basal cells in SqCC contribute to the etiology of ECM-High matreotype tumors. A specific association of IPF with SqCC has been noted in the emergence of squamous metaplasia in fibrotic lung which can then develop into SqCC, as well as the higher incidence of SqCC than adenocarcinoma in IPF patients who develop NSCLC [135, 141–143]. In addition, lung cancer survival is poorer in IPF patients compared with the general population [142]. Our data indicates that a subset of poor-prognosis SqCC patients have hyperactivated fibrogenic signaling mimicking that seen in IPF. This also raises the notion that ECM remodeling, through aging or IPF mechanisms, may contribute to a field of cancerization that primes the lung tissue for accelerated tumorigenesis [144, 145]. These data also suggests that these patients may benefit from anti-stromal therapies targeting these pathways to disrupt the cycle of ECM deposition, fibrogenesis, and the corollary effects on tumor progression.

Anti-stromal therapies are showing considerable promise in the treatment of highly desmoplastic tumors such as pancreatic ductal adenocarcinoma [146], a solid tumor type in which our ECM-High matreotype is also prognostic. The anti-stromal IPF treatment nintedanib, which targets FGF, VEGF, and PDGF signaling, has been approved as a second-line therapy in lung adenocarcinoma as it has demonstrated greater clinical benefit in adenocarcinoma than in the SqCC histological subtype. However, nintedanib has shown some in vitro efficacy in SqCC models albeit lower than in adenocarcinoma models [147] and modest, but significant improvements in progression-free survival and disease control in human SqCC [40, 148]. Our data suggests that correct patient selection would be critical since, unlike the ECM-Low SqCC, the ECM-High subset of SqCC would be the subset most likely to benefit from this treatment. Therefore, with further refinement, matrisomal profiling of SqCC may be used as a companion biomarker for anti-stromal efficacy, and matrisomal subtyping should be considered as an inclusion criterion in the design of future trials. Furthermore, our data suggests that ongoing clinical trials targeting pro-fibrogenic signaling in FGFR-amplified or -mutant SqCC, including those ongoing trials for AZD4547 (FGFR1,2,3; NCT01824901, NCT02154490), lucitanib (FGFR1,2; VEGFR1,2,3, CSF1; NCT012109016), BAY1163877 (NCT01976741), the pan-TKI dovitinib (FGFR + VEGFR, NCT01861197), as well as the use of the approved agent Nintedanib (VEGFR, FGF, PDGFR) [4] should consider using matrisomal subtyping, rather than FGF status alone as an inclusion criteria.

Our data predict that the core matrisome elements within FGFR-wildtype ECM-High matreotype tumors can signal through integrins, FLT4, and PDGFRB on fibroblasts to induce pro-fibrogenic signaling that promotes cancer cell growth, migration, and invasion. Therefore, FGF-wildtype ECM-High tumors may also benefit from these anti-stromal therapies. Furthermore, the association of our ECM-High matreotype with cisplatin resistance, potentially via integrin-mediated induction of cisplatin resistance mediator p21 [108, 109], also suggests that a co-targeting approach of anti-stromal therapies together with standard-of-care platinum doublet therapies may synergize to augment and potentially restore cisplatin sensitivity. Together, these findings indicate that matreotyping SqCC tumors may be useful in identifying those patients with ECM-High tumors who are likely to benefit from stromal co-targeting therapies.

While this study has yielded clinically actionable insights from whole-matrisomal profiling of tumors, it has limitations. The penalized regression modeling approach used to identify the key core matrisomal components defining SqCC implemented parameters that minimized the model error while incorporating a reasonable number of matrisomal genes. However, in balancing model complexity and accuracy, additional matrisomal genes that were less predictive were not included in the final model, yet these may still have some biological or clinical importance in SqCC tumorigenesis. This is particularly the case for highly correlated core matrisomal components, where one ECM component included in the model may capture the predictive power of multiple co-linear ECM components. In addition, our bioinformatic approaches utilized separate tissue sources for the scRNAseq, where SqCC tumor data is particularly limited, and the bulk transcriptomic/proteomic data (RNAseq, whole exome sequencing and RPPA proteomic), making it difficult to discern the precise cell of origin for ECM components. Furthermore, from the analysis of multi-omics data, it is not possible to discern the mechanistic contributions of the ECM component itself, from the exact cell type that produces it, when examining associations with patient outcome. This is in line with recent studies showing that core matrisomal gene expression signatures can robustly recapitulate specific cellular phenotypes [41]. Key ECM components in SqCC risk and prognosis identified in this study have been shown to be expressed by specific subtypes of stromal cells as well as cancer cells and so it is possible that clinical associations identified for matrisomal genes reflect the effects of these cell types more broadly. For example, cancer-associated fibroblasts have also been associated with lung cancer prognosis in adenocarcinoma [116, 149] and NSCLC generally [105, 117, 150], although their significance in SqCC prognosis remains unclear. This in silico analysis will require experimental interrogation to dissect the function of key ECM remodeling features. In particular, functional studies will be required to validate the mechanistic roles of individual matrix components in SqCC prognosis as distinct from effects of cellular subtypes that simply express these matrix components. For example, murine models in which cancer cells are orthotopically injected together with cancer-associated fibroblasts expressing high and low levels of key ECM genes will be fundamental first steps to establishing the functional importance of these ECM components in accelerating tumor progression and metastatic dissemination. Further in vitro studies using cell co-cultures in organotypic matrices will also be critical to dissect signaling mechanisms driving treatment response and metastasis.

## Conclusions

This comprehensive analysis of the SqCC matrisomal landscape has defined key matrisomal components associated with an increased risk of developing lung cancer and of developing aggressive treatment-refractory disease. Through combining multi-omics datasets, we hypothesize that enriched elements of the core matrisome amplify fibrogenesis and activate intracellular signaling networks that support metastatic dissemination. Tumor ECM subtyping has revealed a subset of SqCC patients with the worst prognosis whose tumors are likely to respond well to treatment with existing repurposed anti-stromal therapies in combination with standard-of-care therapy. These findings highlight the importance of considering the ECM profile of SqCC as part of a precision medicine framework to improve the outcome of SqCC patients.

## Supplementary Information

**Additional file 1: Supplementary Materials and Methods**. **Figure S1.** The extracellular matrix is significantly dysregulated in tumor compared with non-tumor tissue in SqCC. **Figure S2.** ECM changes associated with increased lung cancer risk and premalignant progression. **Figure S3.** A) tSNE plot visualization of the expression scores for matrix risk signature genes with positive (B) and negative (C) odds ratios for different cell types (A) in SqCC scRNAseq data presented in Fig. 2D. D) ROC analysis of the minimum matrix risk signature distinguishing progressive from regressive premalignant lesions. E) Matrix risk score at age at diagnosis in the TCGA cohort. Blue line shows linear regression with standard error indicated by grey shading. p=0.00073, r=-0.23, Spearman’s correlation. **Figure S4.** The ECM-High matreotype is associated with poor prognosis. A-B) Relative Cluster Stability Index (A) and p-values (B) for different cluster numbers confirm the presence of three major matreotypes in SqCC. C) The correlation plot for samples corresponding to the heatmap in Fig. 3A). D) Heatmap of marker genes for the ECM-High and ECM-Low matreotypes in the TCGA cohort. E-F) Matreotype association with survival in early stage (E, Stage I and II) patients (Ci) and late stage patients (F, Stage III and IV) patients in the TCGA LUSC cohort. G) Recurrence-free survival for ECM-High and ECM-Low matreotypes in the UHN cohort of early-stage tumors (log-rank p=0.19). H) Representative H&E and picrosirius red-stained tissue microarray cores corresponding to high (upper panel) and low (lower panel) picrosirius-red-stained tumors. Scale bar = 500 μm. I-J) Overall survival of patients according to picosirius red staining for tumors across all stages (I, univariate cox-proportional hazards model HR = 1.76 [1.04-2.98], p=0.035; multivariate coxproportional hazards model HR = 1.87 [1.09-3.2], p=0.023) and stages I and IIA only (J, univariate cox-proportional hazards model HR = 2.4 [1.3-4.4], p=0.0051; multivariate cox-proportional hazards model HR = 2.4 [1.3-4.4], p=0.0051)). Multivariate cox-proportional hazards models include stage as a clinical covariate. **Figure S5.** A) Mutational frequency plot showing no enrichment of driver mutations or FGFR amplification in each matreotype. B) Mutational frequency plot showing the top mutated genes differentially enriched in the ECM-High vs ECMLow matreotype tumors. C) Disease-specific survival of adenocarcinoma samples assigned to SqCC matreotypes shows no significant association of SqCC matreotypes with prognosis in the adenocarcinoma subtype (log-rank p=0.74). D-E) The hazard ratios for Disease-specific survival (D) and Progression-Free Survival (E) of ECM-High pan-cancer tumors compared with ECM-Low matreotypes in multiple cancer types. Red dots indicate tumor types with significant hazard ratios for the ECM-High matreotype compared with the ECM-Low matreotype. Hazard ratios and confidence intervals are in Additional File 1: Table S8. **Figure S6.** SqCC Matreotypes have distinct immunological ecosystems. **Figure S7.** Extracellular Matrix components contribute to signaling pathways associated with prognosis. **Figure S8.** ECM-driven integrin signaling is associated with EMT and fibroblast activation in the ECM-High matreotype. **Figure S9.** The poor prognosis matreotype overlaps with ECM remodeling in Idiopathic Pulmonary Fibrosis. **Table S1.** Matrix Risk Score. **Table S2.** Pan-Cancer Risk Score Results. **Table S3.** Optimized Minimum Matrisomal Linear Model for Premalignant Progression. **Table S4.** Clinicodemographic Features of the TCGA Matreotypes. **Table S5.** Clinicodemographic Features of the NCI-MD cohort. **Table S6.** Clinicodemographic Features of the Tissue Microarray Cohort. **Table S7.** Cox Proportional Hazards Model Analysis of Picrosirius Red stained TMAs. **Table S8.** Pan-Cancer Prognostic Matreotype Results.

**Additional file 2: Table S1.** Differentially Expressed Hallmark Pathways in ECM-High vs ECM-Low Tumors. **Table S2.** Differentially Expressed MSigDb C2 Pathways in ECM-High vs ECM-Low Tumors. **Table S3.** Ligand-Receptor Interactions in ECM-High vs ECM-Low Tumors.

## Data Availability

The NCI-MD cohort RNAseq data analyzed in this study are accessible at the NCBI GEO website under the accession number GSE201221 (https://www.ncbi.nlm.nih.gov/geo/query/acc.cgi?acc=GSE201221) [71]. Publicly available TCGA LUSC and LUAD RNAseq, WES and CNV data are available from Broad GDAC Firehose (https://gdac.broadinstitute.org). Publicly available Pan-Cancer RNAseq and survival data is available from Genomic Data Commons repository (https://gdc.cancer.gov/about-data/publications/pancanatlas). Publicly available TCGA RPPA data is available from the TCPA Portal (https://tcpaportal.org/tcpa/download.html). Publicly available data from the UHN cohort is available in the Gene Expression Omnibus repository, GSE50081 (https://www.ncbi.nlm.nih.gov/geo/query/acc.cgi?acc=GSE50081). Publicly available RNAseq of squamous carcinoma in situ is available in the Gene Expression Omnibus repository, GSE108124 (https://www.ncbi.nlm.nih.gov/geo/query/acc.cgi?acc=GSE108124). scRNAseq data are available from https://lambrechtslab.sites.vib.ve/en/data-access. RNAseq data of the aging lung is available in the Gene Expression Omnibus repository, GSE165192 (https://www.ncbi.nlm.nih.gov/geo/query/acc.cgi?acc=GSE165192).

## Abbreviations

NSCLC: Non-small cell lung cancer
SqCC: Squamous NSCLC
