# Supplementary material for: Extracellular matrix profiles determine risk and prognosis of the squamous cell carcinoma subtype of non-small cell lung carcinoma
Source: Genome Med. 2022 Nov 21;14:126. doi: 10.1186/s13073-022-01127-6 (PMC9677915; doi:10.1186/s13073-022-01127-6)
Supplement: Supplementary file 1 — Additional file 1: Supplementary Materials and Methods. Figure S1. The extracellular matrix is significantly dysregulated in tumor compared with non-tumor tissue in SqCC. Figure S2. ECM changes associated with increased lung cancer risk and premalignant progression. Figure S3. A) tSNE plot visualization of the expression scores for matrix risk signature genes with positive (B) and negative (C) odds ratios for different cell types (A) in SqCC scRNAseq data presented in Fig. 2D. D) ROC analysis of the minimum matrix risk signature distinguishing progressive from regressive premalignant lesions. E) Matrix risk score at age at diagnosis in the TCGA cohort. Blue line shows linear regression with standard error indicated by grey shading. p=0.00073, r=-0.23, Spearman’s correlation. Figure S4. The ECM-High matreotype is associated with poor prognosis. A-B) Relative Cluster Stability Index (A) and p-values (B) for different cluster numbers confirm the presence of three major matreotypes in SqCC. C) The correlation plot for samples corresponding to the heatmap in Fig. 3A). D) Heatmap of marker genes for the ECM-High and ECM-Low matreotypes in the TCGA cohort. E-F) Matreotype association with survival in early stage (E, Stage I and II) patients (Ci) and late stage patients (F, Stage III and IV) patients in the TCGA LUSC cohort. G) Recurrence-free survival for ECM-High and ECM-Low matreotypes in the UHN cohort of early-stage tumors (log-rank p=0.19). H) Representative H&E and picrosirius red-stained tissue microarray cores corresponding to high (upper panel) and low (lower panel) picrosirius-red-stained tumors. Scale bar = 500 μm. I-J) Overall survival of patients according to picosirius red staining for tumors across all stages (I, univariate cox-proportional hazards model HR = 1.76 [1.04-2.98], p=0.035; multivariate coxproportional hazards model HR = 1.87 [1.09-3.2], p=0.023) and stages I and IIA only (J, univariate cox-proportional hazards model HR = 2.4 [1.3-4.4], p=0.0051; [file 13073_2022_1127_MOESM1_ESM.pdf]

Additional File 1

Extracellular Matrix Profiles Determine Squamous Non-Small Cell Lung Cancer Risk and Prognosis

Parker *et al.*

Supplementary Materials and Methods

RNA Sequencing

Lung tumor and adjacent non-tumor tissue samples were collected as part of the NCI-University of Maryland Study according to protocols approved by the institutional review board (OH98-C-N027, National Cancer Institute, USA) and processed as described previously (1,2). Briefly, total RNA was extracted from the tissues and libraries suitable for paired-ended sequencing were generated using Illumina's TruSeq Stranded Total RNA Library Prep Kit. RNA samples were pooled and sequenced on a HiSeq. The sequencing quality of each sample was assessed using FastQC (3). Read- and alignment-level quality were assessed using FastQ Screen (4), Preseq (5) Picard tools, RSeQC (6) and QualiMap (7). Reads were trimmed for adapter sequences and low-quality bases using Cutadapt (8) prior to alignment against the human reference genome, hg19, using STAR (9) in two-pass mode with GENCODE v19 annotation (10). Gene expression levels were quantified using RSEM (11). Batch correction was performed using the Combat algorithm from the SVA package (12).

Bulk RNAseq Analysis of Squamous Cell Carcinomas

RSEM-quantified RNASeq abundances and relevant clinical information from publicly available datasets were accessed according to Table 1. Gene abundance accessed as count data were converted to counts per million before being filtered to remove genes with counts less than 10 in more than two samples using the EdgeR package (13). Read counts were TMM normalized (EdgeR package (13)). Normalized RNAseq abundances were log2 transformed.

Matrisomal genes were defined as core matrisome or matrisome-associated according to Naba et al. (2012) (14). Principal Component Analysis of tumor compared with non-tumor tissue was performed using the prcomp algorithm and default settings in base R. Conversions between gene symbols and species were performed using the BiomaRt package (15). Differential gene expression analysis between tumor and non-tumor tissue was performed by fitting a linear model (lmFit algorithm with trend = TRUE, limma package) and calculation of differential expression by empirical Bayes moderation of the standard error to a global value (eBayes algorithm with default settings, limma package) (16). In datasets with highly variable library sizes, differential

gene expression analysis was performed using the voom algorithm (default settings, Limma package (16)). Heatmaps of gene expression matrices were generated using the ComplexHeatmap package (17) for visualization.

Matrix Risk Signature Generation

Risk signature feature selection was performed on differentially expressed core matrisomal genes between tumor and non-tumor tissue in the TCGA LUSC dataset. Genes were selected based on their association with tumor compared with non-tumor tissue using a penalized logistic regression implemented using the glmfit function from the glmnet package (18), to account for the high degree of correlation of some matrix genes. Z-scaled RNAseq expression data were initially partitioned 80% to 20% into training and test datasets, respectively using the createDataPartition algorithm of the caret package and the model was developed using training data only. Penalized logistic regression was implemented to refine the matrix risk signature to include only those matrisomal genes that most significantly discriminated between tumor and non-tumor tissue while ensuring that the model was not over-fitted to the data. The penalized logistic regression model was developed on the training dataset and was considered most appropriate due to the large variance in the ordinary least squares estimator due to some highly correlated matrix genes. The coefficient of shrinkage (lambda) was calculated to balance the number of predictors against accuracy and was chosen as the value within one standard error of the lambda that minimized the cross-validation prediction error rate, implemented using the cv.glmnet algorithm. Alpha was set to 0.5 to apply Elastic Net regression. Elastic net regression was selected over lasso regression as elastic net regression balances the minimization and elimination of poorly -predicting variables, while lasso regression eliminates variables and can remove too many variables, causing poor generalization of the model (18). As a result, elastic net regression retains as many ECM genes in the signature required to accurately fit the data, with more ECM genes to enable the model to perform well when applied to independent datasets. The final model was applied to the test data where it gave 100% accuracy of predicting tumor and non-tumor tissues. To generate a matrix risk score and for visualization purposes, Odds ratios were calculated using Firth's correction using the logistf algorithm from the logistf package. The matrix risk score for each

sample was summed as the product of the log(Odds Ratio) (**Supplementary Table 1**) and the expression value for each z-scaled gene expression (Equation 1).

$$Matrix\ Risk\ Score = \sum_{i=1}^{n} z_i \beta_i \qquad \text{(Equation 1)}$$

Where i = gene in the matrix risk signature of length n

z_i = z-scaled gene expression of gene i

β_i = log (Odds Ratio) of gene i

Matreotype Identification

Identification of matreotypes was performed using monte-carlo reference-based consensus clustering implemented through the M3C command using the K-means clustering algorithm (default settings, M3C package (19)) applied to the TCGA expression matrix of significantly differentially expressed core matrisomal genes from tumor and non-tumor tissue. We used this algorithm to accurately determine the optimal cluster number and avoid overfitting of the data by simulating a null distribution of stability scores for a range of cluster numbers (K) and correcting the inherent bias of consensus clustering towards high K values. Cluster number was determined as the number of clusters to give a maximal Relative Cluster Stability Index (RCSI), a Monte -Carlo p-value less than 0.05 and a minimal Proportional of Ambiguous Clustering (PAC) Score (19). Cluster assignments were manually checked by Principal Component Analysis (PCA). Clustering statistics (silhouette width, Dunn index) were calculated by the cluster.stats algorithm of the fpc package.

Centroids for each matreotype were calculated on the TCGA LUSC dataset as the mean z-scaled expression level for those genes used to identify the matreotypes (i.e. significantly differentially expressed core matrisomal genes between tumor and non-tumor tissue). Matreotypes were assigned as the matreotype that gave the minimal Euclidean distance between each sample and the matreotype centroids. The association of matreotypes with categorical clinicodemographic information was assessed using the Fisher's exact test. Survival associations were assessed using the Survival and Survminer packages. Hazard ratios were calculated using the coxph function from the Surv package with corrections for stage and age, which were clinical covariates significantly associated with survival in univariate analysis. Gender, smoking status

and pack years were clinical covariates not found to be significantly associated with survival in a univariate analysis so were not included in the multivariate model.

Matreotype associations with driver mutations were assessed using maftools. FGFR copy number variation analysis was performed on TCGA CNV data using probes mapped to the segment containing the FGFR cluster at chromosome 8p11.23 (Hg18; 38 387 813-38 445 509). Segment mean values < -0.7 were considered to have copy number losses and values > 0.7 were considered copy number gains, with values of -0.7 to 0.7 considered to be wild type for FGFR amplification. The association of FGFR amplifications with ECM-High and ECM-Low matreotypes were assessed by Fisher's exact test.

Pathway Analyses

Pathway analysis was performed in R (v3.6.3) on the 50 hallmark pathways and C2 oncogenic pathways described in the molecular signatures database. Pathway signatures were accessed in R using the mSigDb package (https://davislaboratory.github.io/msigdb). To assign pathway activity estimates for each Hallmark and C2 oncogenic pathway to each sample, we performed Gene Set Variation Analysis by implementing the gsva algorithm with default settings (GSVA package (20)). Differential enrichment of these pathway activity estimates in the different matreotypes were tested by fitting a linear model (lmFit algorithm with default settings, limma package) and calculation of differential expression by empirical Bayes moderation of the standard error to a global value (eBayes algorithm with default settings, limma package) (16).

Ligand Receptor Interaction Analysis

Genes within the RNAseq datasets were annotated as ligands and receptors based on the curated database of human ligand-receptor pairs previously published by Ramilowski et al. (21) based on supporting literature. Only ligands corresponding to core matrisome genes as defined by Naba et al. (2012) (14) were retained for further analysis. We used this database to identify receptors that the core matrisomal components interact with to influence cell signaling processes within the tumor microenvironment. The strength of these ligand receptor interactions were calculated

as an interaction score, based on the expression level of the core matrisomal ligand and its cognate receptor, as described previously (22). Mathematically, the interaction score of each ligand-receptor pair was calculated as the product of expression values from the ligand (i.e. core matrisomal gene) and its cognate receptor in each sample, (Equation 2).

$$Interaction\ Score = R_i L_i \qquad \text{(Equation 2)}$$

Where R_i = z-scaled gene expression of receptor i

L_i = z-scaled gene expression of ligand i

To provide a broad view of enriched receptor classes in each matreotype, receptors were manually annotated into receptor classes based on literature searches. Interaction scores for each receptor class were then calculated as the maximum interaction score for that receptor class.

Differential enrichment of ligand-receptor interaction scores in the different matreotypes were tested by fitting a linear model (lmFit algorithm with default settings, limma package) and calculation of differential expression by empirical Bayes moderation of the standard error to a global value (eBayes algorithm with default settings, limma package) (16). Differentially enriched ligand-receptor type scores in the ECM-High matreotype compared with the ECM-Low matreotype were then visualized as a circos plot (circos algorithm, circlize package (23)). To examine the association of differentially enriched ligand-receptor pairs with downstream signaling pathways, receptors were annotated with the Hallmark signatures (MSigDb) signatures that they are represented in and ligand-receptor interaction scores were aggregated into ligand-Hallmark signature scores using the maximum ligand-receptor interaction score for each Hallmark signature. For a focused analysis of how the core matrisome of these ECM subtypes are associated with Hallmark pathways, only those ligands (i.e. core matrisomal genes) that were differentially expressed in the ECM-High matreotype compared with the ECM-Low matreotype were included in this analysis.

Cellular Composition Analysis

Two main approaches were implemented to identify enriched cell types in SqCC matreotypes using datasets described in Table 1. Deconvolution of bulk TCGA LUSC RNAseq data into NSCLC epithelial, fibroblast and endothelial cell types was performed using CibersortX and the signature matrix derived from scRNAseq analysis of NSCLC tumours published by Lambrechts et al. (2018) (24). CibersortX was run in absolute mode with batch correction and 100 permutations. Quantile normalization was disabled as recommended for RNAseq data. To assess differential enrichment of these cell types in normal tissue and tumour matreotypes, CibersortX enrichment scores were tested by fitting a linear model (lmFit algorithm with default settings, limma package) and calculation of differential enrichment between groups by empirical Bayes moderation of the standard error to a global value (eBayes algorithm with default settings, limma package) (16).

To infer the relative enrichment of immune cells within the tumor microenvironment, immune cell type scores were assigned to each sample by applying NSCLC-specific immune cell signatures developed by Faruki et al. (2017) (25) to bulk TCGA LUSC RNAseq data using Gene Set Variation Analysis (gsva algorithm, GSVA package, default settings). Differential enrichment of immune cell type scores in the ECM-High vs ECM-Low matreotypes was assessed by Mann-Whitney U-test and in more than two groups by the Kruskal Wallis tests. For multiple comparisons, p-values were corrected using the Benjamini-Hochberg method.

Single Cell RNAseq Analysis of Squamous Cell Carcinoma

Raw gene expression matrices and cellular metadata including cell type assignments from scRNAseq data of NSCLC samples were obtained from (24,26) and processed as described. Briefly, scRNAseq expression processing was performed in the Seurat package (v4.0.1). Cells with fewer than 201 UMIs and over 6000 or below 101 expressed genes, or over 10% UMIs derived from the mitochondrial genome were removed. Gene expression matrices were normalized to total cellular read count and mitochondrial read count as implemented by Seurat's normalize and scale functions. Variably expressed genes were selected as having a normalized expression between 0.125 and 3 and variance exceeding 0.5. To reduce dimensionality of the dataset,

variably expressed genes were summarized by Principal Component Analysis (PCA) and the first 8 principal components were further summarized using tSNE dimensionality reduction using the default settings of the RunTSNE function. Cell clusters were then annotated using the cluster annotations assigned by (24). Cells corresponding to SqCC tumors were retained for further analysis. Cell type scores for ECM genes were assigned using the AddModuleScore function (Seurat package, default settings). Matrix risk scores were calculated on the z-scaled gene expression matrix for each cell type using Equation 1 as described above (Matrix Risk Signature section).

Fibrosis Score

In order to characterize the extent to which SqCC ECM remodeling overlaps with that in Idiopathic Fibrosis we calculated an IPF fibrosis score for each TCGA sample based on the expression of differentially expressed genes in in human IPF lungs compared with normal lungs described by McDonough et al. (2018) (27). Z-scaled RNAseq gene expression data was scaled to between 0 and 1 based on the expression range for that particular gene. This normalized gene expression was then weighted by positive 1 for genes upregulated in IPF lungs, and -1 for genes downregulated in IPF lungs. The IPF fibrosis score was calculated as the sum of these weighted gene scores (Equation 3).

$$IPF\ Fibrosis\ Score\ = \sum_i^n w_i z_i \qquad \text{(Equation 3)}$$

Where w_i = 1 for gene upregulated in IPF lungs and -1 for genes downregulated in IPF lungs for gene i in the n gene signature

Z_i = scaled gene expression for gene i in the n gene signature

Transcription Factor Enrichment Analysis

Transcription factor analysis on core matrisomal correlation clusters was performed using the chEA transcription factor targets database using the chEA3 web interface (28). which mines a database of CHIP-based experimental results and scores gene sets for transcription factor associations based on ENCODE Chip-Seq, publicly available CHIP-Seq data describing binding site proximity as well as the co-expression of genes in the GTeX and ARCHS4 datasets. As the transcription factor regulation of many ECM genes have not yet been comprehensively characterized, integrating multiple CHIP databases with expression analysis in this approach expands the scope for

identifying upstream regulators of ECM gene expression. Gene lists corresponding to each core matrisomal correlation cluster were uploaded to the chEA3 server and the integrated mean rank for each transcription factor was calculated for each correlation cluster.

Picrosirius Red Staining and Quantitation of Tissue Microarrays

Each core of the tissue microarray was assessed for tumor tissue by a registered clinical pathologist (Dr W. Cooper). Paraffin-embedded tissue microarrays were cut into 4μm sections, rehydrated and stained with 0.1% picrosirius red (Poly-sciences) for fibrillar collagen according to manufacturer's instructions. Slides were counterstained with haematoxylin, before dehydration and cover slipping. Polarised light tiled imaging was performed on a Leica DM 6000 microscope fitted with posterior and anterior polarising filters at 20x magnification. Individual cores were exported from Qupath (0.2.3) and quantified for picrosirius red birefringence using an in-house script as previously published (69). Quantitative measurements of fibrillar collagen signal (red) were performed using thresholds of Hue 0-27 and Brightness 10-255 and total tissue area was normalized against the tissue area defined with a saturation threshold of 59-255. The total red area was normalized to the total tissue area to account for spaces within the tissue corresponding to vessel and airway lumens.

To assess the association of picrosirius red staining with survival, the maximum total red signal normalised to tissue area across three to five tumor cores was calculated per patient. Patients were stratified into high and low groups by the 60th percentile. This picrosirius red signal was combined with clinicodemographic information and the association with survival was assessed using Cox proportional hazards model (coxph algorithm, survival package) in R (v3.6.1). To identify clinical co-variates that are significantly associated with survival in this cohort, age, gender, stage and lymph node positivity were individually assessed for association with outcome in a univariate cox-proportional hazards model. Stage and lymph node positivity were identified as being significantly associated with survival, with the latter concordant with a stage IIA and higher diagnosis. For multivariate analysis of picrosirius red signal with overall survival, stage and lymph node positivity were included as clinical covariates. For visualisation purposes, Kaplan-meier plots were generated using the survminer survival packages.

Additional Figures

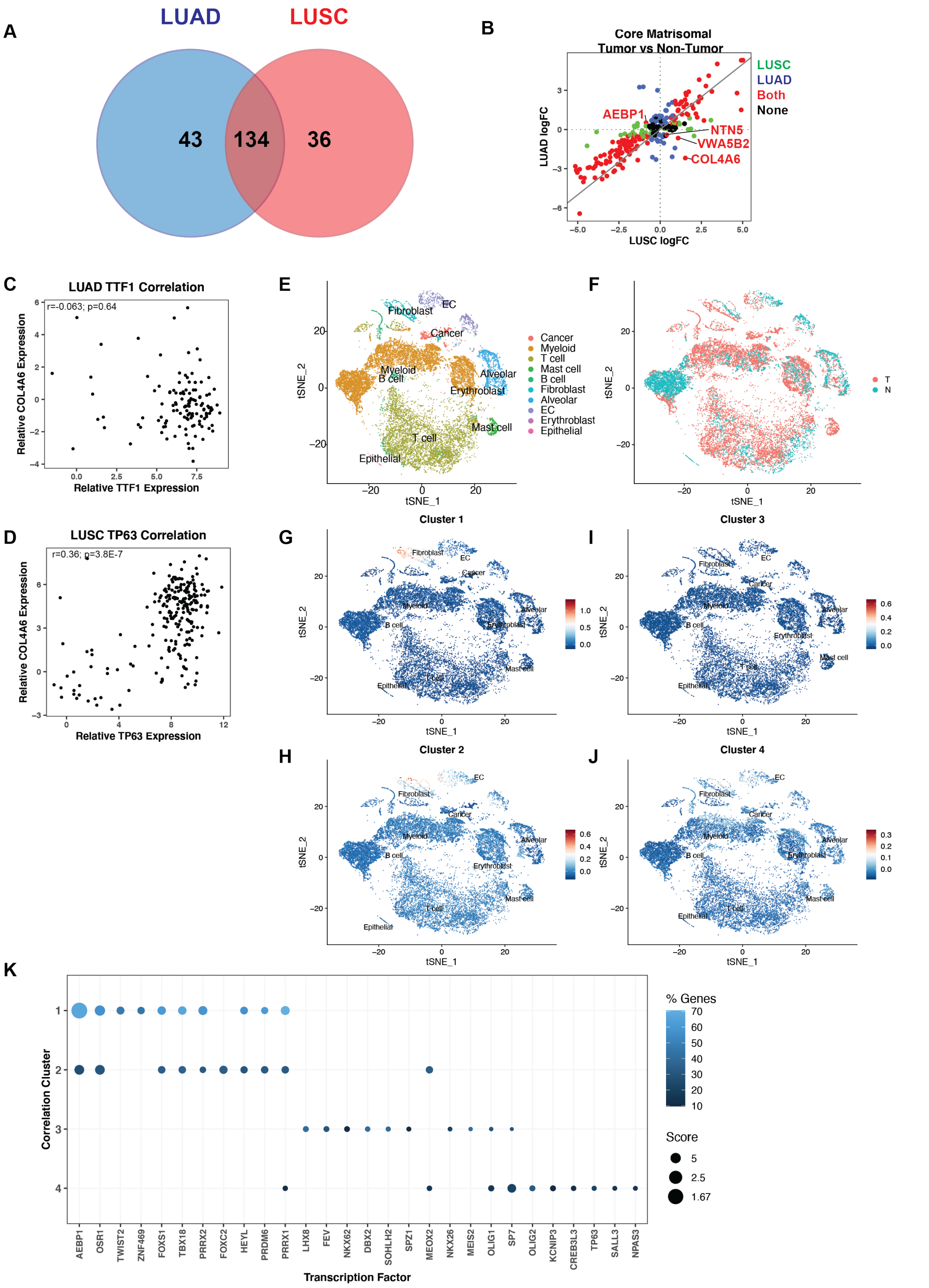

Additional File 1: Figure S1. The extracellular matrix is significantly dysregulated in tumor compared with non-tumor tissue in SqCC. A) Differentially expressed core matrisome genes in adenocarcinoma (LUAD) and squamous NSCLC (LUSC). **B)** Log fold change (logFC) of differentially expressed core matrisomal genes in adenocarcinoma (LUAD) and squamous (LUSC) subtypes of NSCLC. Green dots =

genes significantly differentially expressed in LUSC only; Blue dots = genes significantly differentially expressed in LUAD only; Red dots = genes significantly differentially expressed in both LUAD and LUSC; Black dots = genes not significantly differentially expressed in either LUAD or LUSC; **C and D)** COL4A6 expression relative to TTF1 in LUAD (**C**, r=-0.063, p=0.64) and relative to TP63 in LUSC (**D**, r=0.36, p=3.8E-7). Spearman's correlation test. **E - J)** Expression of correlated clusters in scRNAseq data with **E)** cell type clusters; **F)** tumor (orange, T) compared with non-tumor (blue, N); **G)** correlation cluster 1; **H)** correlation cluster 2; **I)** correlation cluster 3 and **J)** correlation cluster 4. Blue to Red scale indicates expression level with indicating high expression. **K)** Transcription factor enrichment analysis of correlation clusters in Figure 1D and Supplementary Figure 1D). Dot size indicates rank of chEA score (low number = high enrichment strength) and color indicates the percentage of genes in that correlation cluster mapping to that transcription factor.

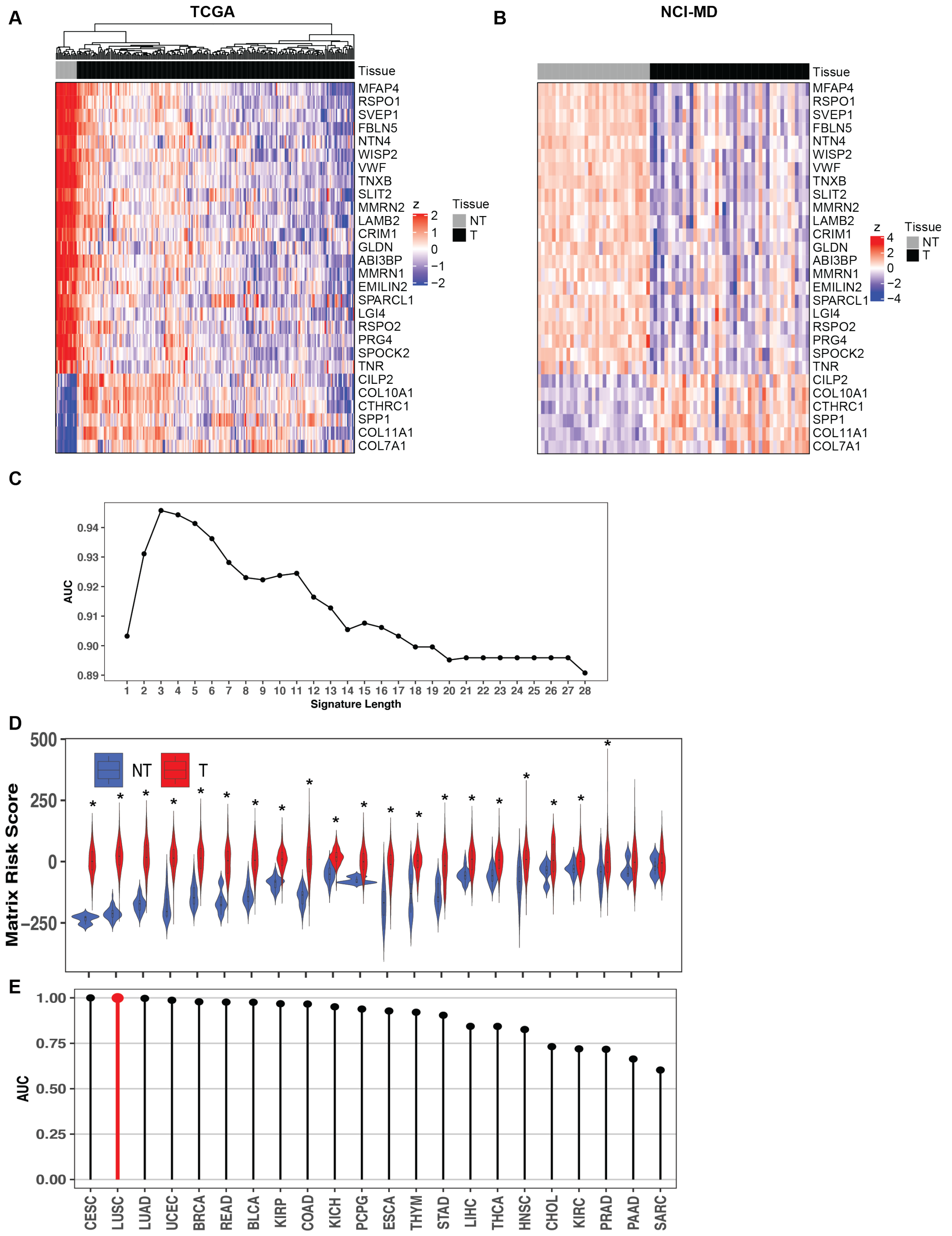

Additional File 1: Figure S2. ECM changes associated with increased lung cancer risk and premalignant progression. A-B) Heatmap of matrix risk genes in SqCC tumors from the TCGA (A) and NCI-MD cohorts (B). **C)** Minimum gene signature length and predictive power of the matrix risk signature in the NCI-MD cohort. **D)** Pan-cancer analysis of the matrix risk score in tumor (T, red) and non-tumor (NT, blue) tissue in multiple cancer types; *p<0.05. Mann-Whitney U-test with Benjamini

Hochberg correction for multiple comparisons. See Additional File 1: Table S2 for statistical analyses**. E)** Corresponding Area Under the Curve for the ROC analysis for the same tumor types. See Additional File 1: Table S2 for ROC analysis data. CESC: cervical squamous cell carcinoma and endocervical adenocarcinoma (CESC); LUSC: lung squamous carcinoma; LUAD: lung adenocarcinoma; UCEC: uterine corpus endometrial carcinoma; BRCA: breast invasive carcinoma; READ: rectum adenocarcinoma; BLCA: bladder urothelial carcinoma; KIRP: kidney renal papillary cell carcinoma; COAD: colon adenocarcinoma; KICH: kidney chromophobe; PCPG: pheochromocytoma and paraganglioma; ESCA: esophageal carcinoma; THYM: thymoma; STAD: stomach adenocarcinoma; LIHC: liver hepatocellular carcinoma; THCA: thyroid carcinoma and HNSC: head and neck squamous cell carcinoma; CHOL: cholangiocarcinoma; KIRC: kidney renal clear cell carcinoma; PRAD: prostate adenocarcinoma; PAAD: pancreatic adenocarcinoma; SARC: sarcoma.

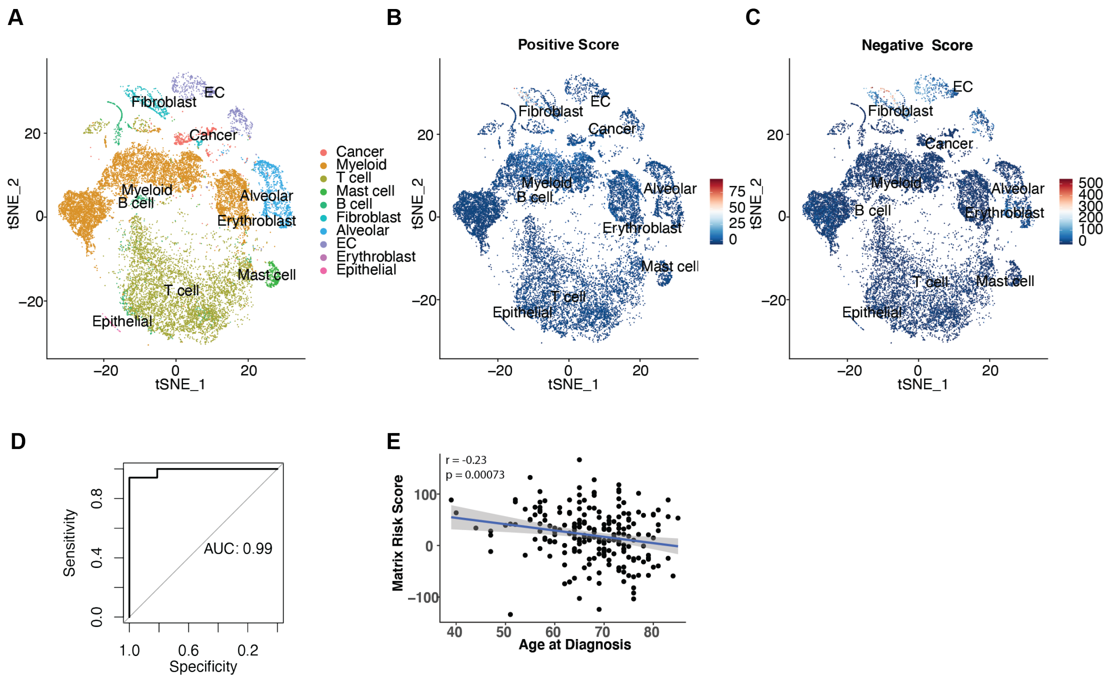

Additional File 1: Figure S3. **A)** tSNE plot visualization of the expression scores for matrix risk signature genes with positive **(B)** and negative **(C)** odds ratios for different cell types **(A)** in SqCC scRNAseq data presented in Figure 2D. **D)** ROC analysis of the minimum matrix risk signature distinguishing progressive from regressive premalignant lesions. **E)** Matrix risk score at age at diagnosis in the TCGA cohort. Blue line shows linear regression with standard error indicated by grey shading. p=0.00073, r=-0.23, Spearman's correlation.

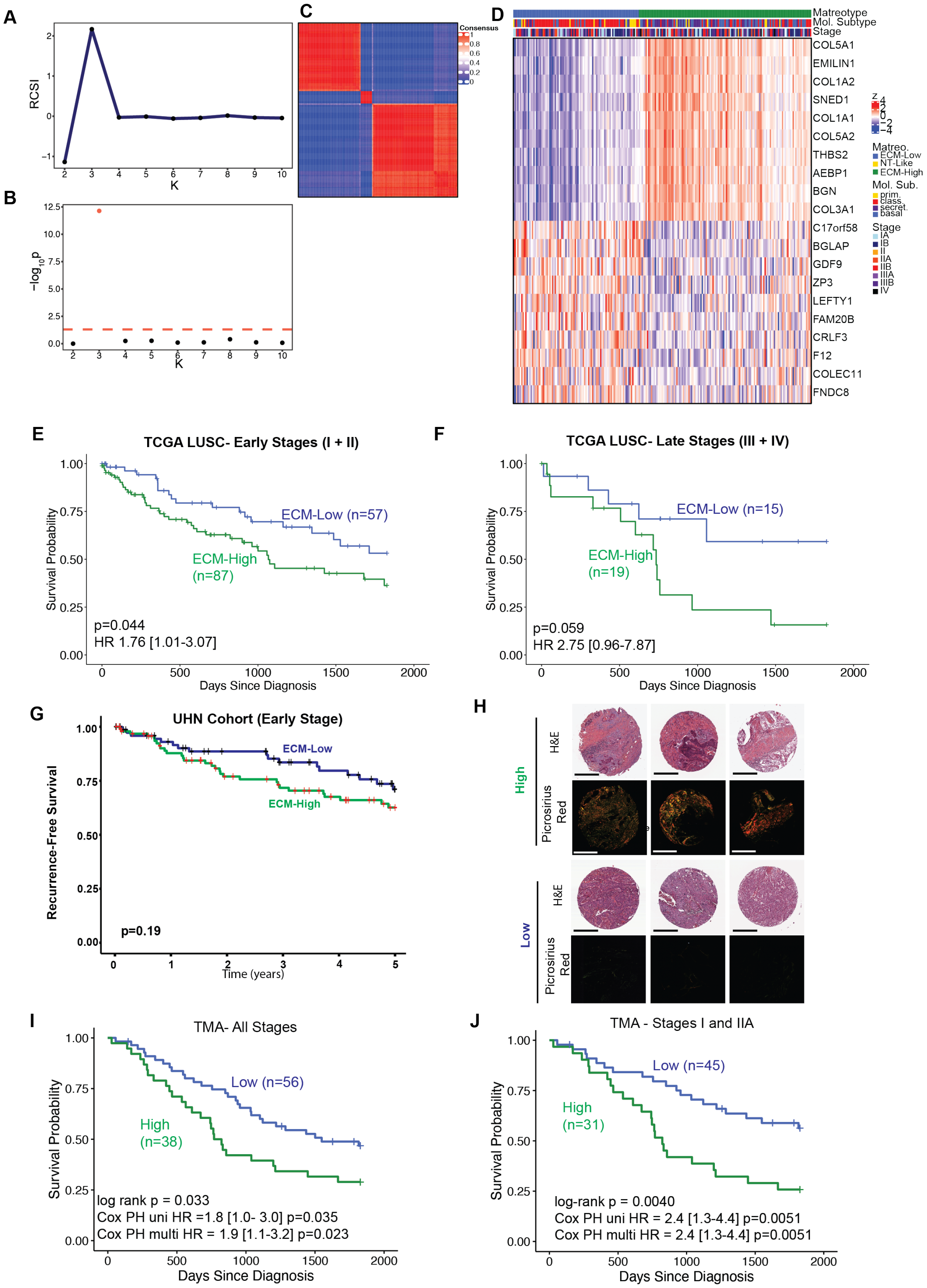

Additional File 1: Figure S4. The ECM-High matreotype is associated with poor prognosis. A-B) Relative Cluster Stability Index **(A)** and p-values **(B)** for different cluster numbers confirm the presence of three major matreotypes in SqCC. **C)** The correlation plot for samples corresponding to the heatmap in Figure 3A). **D)** Heatmap

of marker genes for the ECM-High and ECM-Low matreotypes in the TCGA cohort. **E-F)** Matreotype association with survival in early stage (**E**, Stage I and II) and late stage (**F**, Stage III and IV) patients in the TCGA LUSC cohort.

G) Recurrence-free survival for ECM-High and ECM-Low matreotypes in the UHN cohort of early-stage tumors (log-rank p=0.19). **H)** Representative H&E and picrosirius red-stained tissue microarray cores corresponding to high (upper panel) and low (lower panel) picrosirius-red-stained tumors. Scale bar = 500 µm. **I-J)** Overall survival of patients according to picosirius red staining for tumors across all stages (**I**, univariate cox-proportional hazards model HR = 1.76 [1.04-2.98], p=0.035; multivariate cox-proportional hazards model HR = 1.87 [1.09-3.2], p=0.023) and stages I and IIA only (**J**, univariate cox-proportional hazards model HR = 2.4 [1.3-4.4], p=0.0051; multivariate cox-proportional hazards model HR = 2.4 [1.3-4.4], p=0.0051). Multivariate cox-proportional hazards models include stage as a clinical covariate.

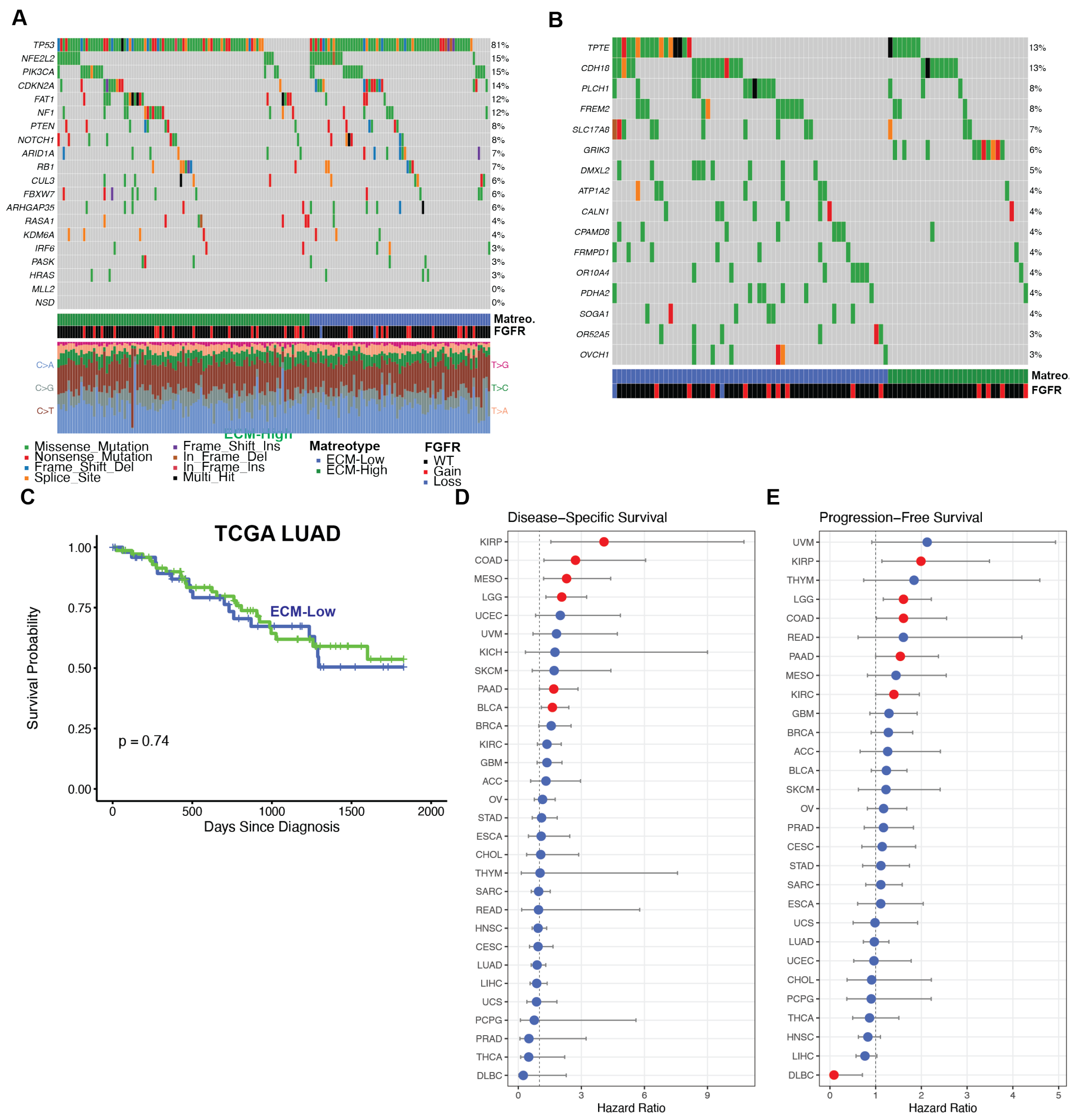

Additional File 1: Figure S5. A) Mutational frequency plot showing no significant enrichment of driver mutations or FGFR amplification in each matreotype. **B)** Mutational frequency plot showing the top mutated genes differentially enriched in the ECM-High vs ECM-Low matreotype tumors. **C)** Disease-specific survival of adenocarcinoma samples assigned to SqCC matreotypes shows no significant association of SqCC matreotypes with prognosis in the adenocarcinoma subtype (log-rank p=0.74). **D-E)** The hazard ratios for Disease-specific survival **(D)** and Progression-Free Survival **(E)** of ECM-High pan-cancer tumors compared with ECM-Low matreotypes in multiple cancer types. Red dots indicate tumor types with significant hazard ratios for the ECM-High matreotype compared with the ECM-Low matreotype. Hazard ratios and confidence intervals are in Additional File 1: Table S8.

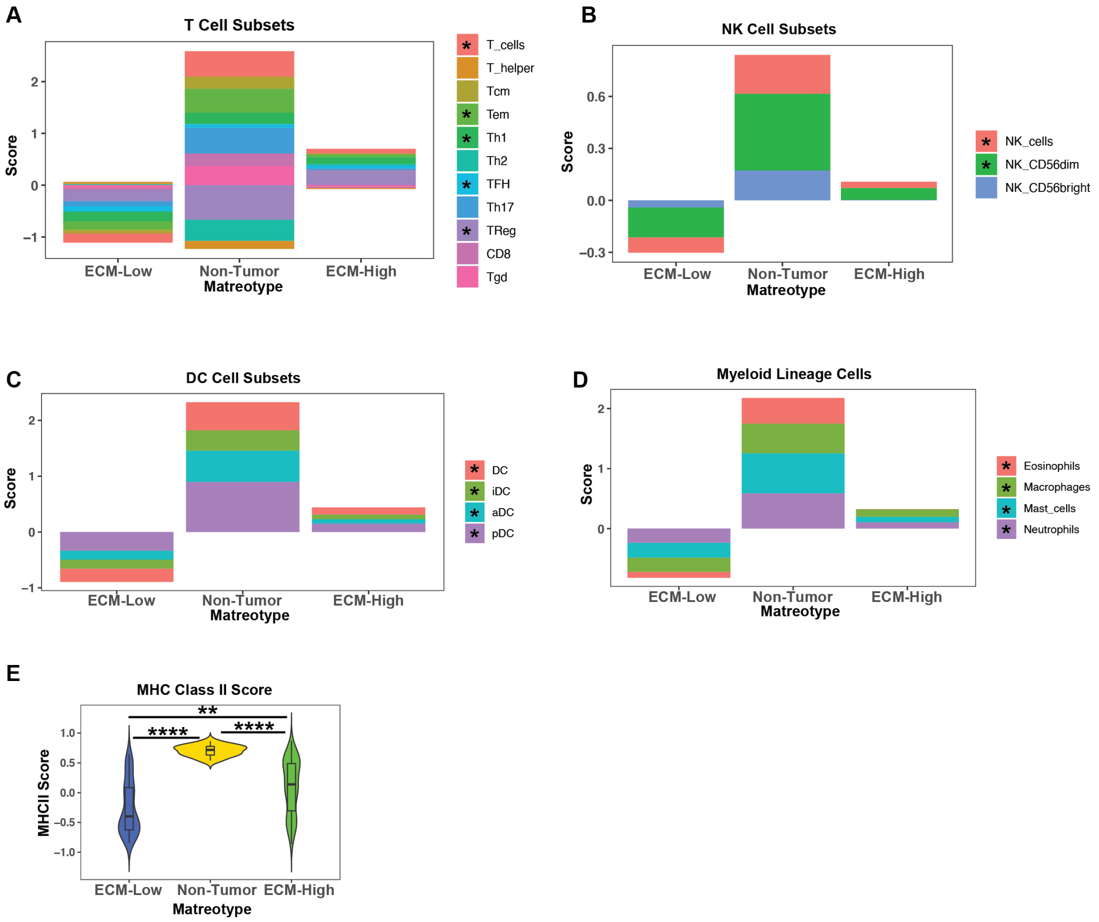

Additional File 1: Figure S6. SqCC Matreotypes have distinct immunological ecosystems. A) Comparison of immune cell type scores in the ECM-High (green), ECM-Low (blue) and non-tumor (yellow) matreotypes for T cells (**A**), NK cells (**B**), Dendritic Cells (**C**) and Myeloid lineage cells (**D**). * indicates $p_{adj}<0.05$ for ECM-High vs ECM-Low for Mann-Whitney U-test with Benjamini-Hochberg adjustment. **E**) MHC Class II expression score in SqCC matreotypes. ECM-High vs ECM-Low p=4.4E-6, ECM-High vs Non-Tumor p=4.0E-7, ECM-Low vs Non-Tumor p=9.2E-9, Mann-Whitney U-test.

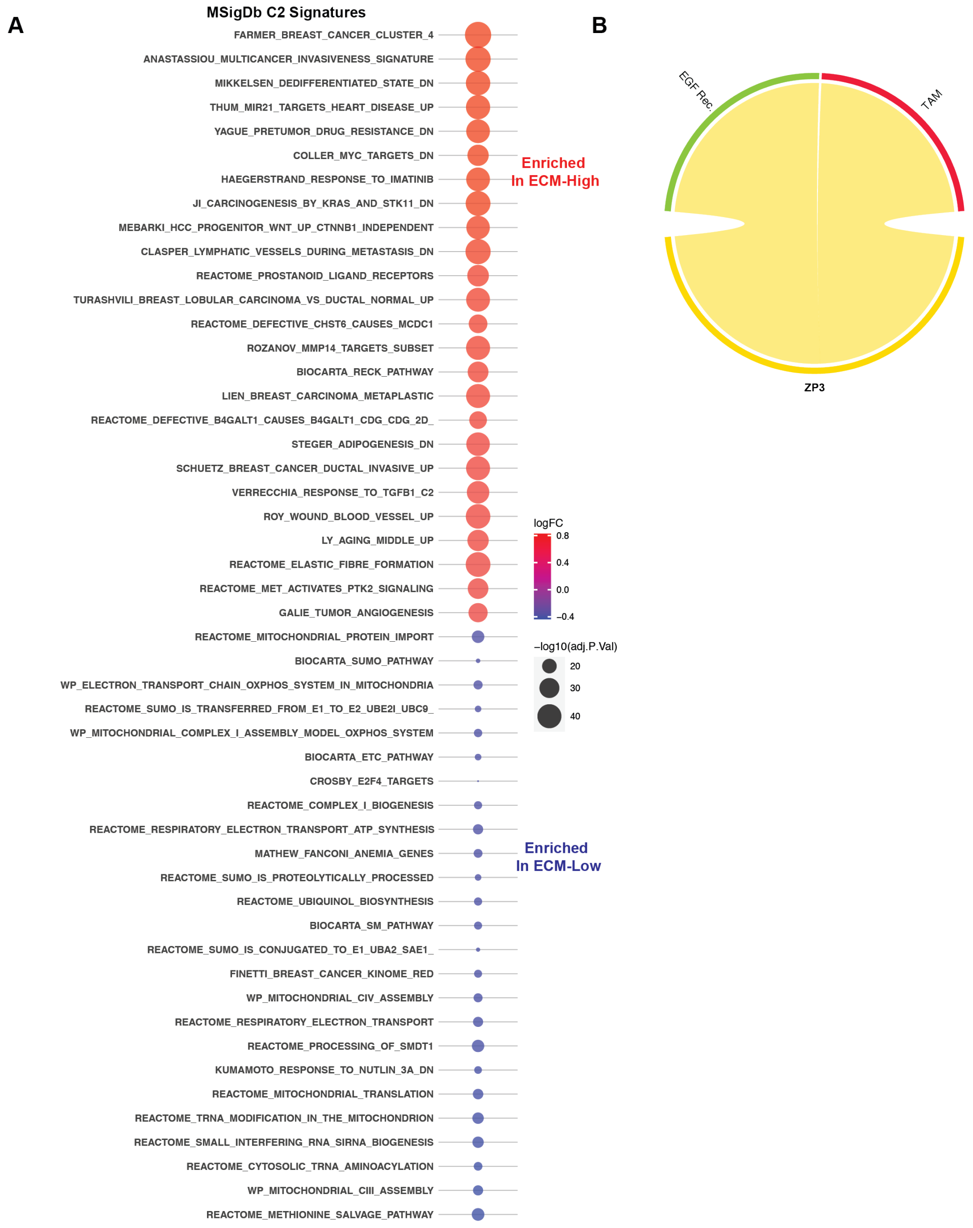

Additional File 1: Figure S7. Extracellular Matrix components contribute to signaling pathways associated with prognosis. A) Top 25 up and down regulated differentially activated oncogenic (MSigDb C2) pathways in the ECM-High matreotype compared with the ECM-Low matreotype. LogFC= log fold change in ECM-High compared with the ECM-Low. **B**) Receptors activated by core matrisomal components (i.e. ZP3) enriched in the ECM-Low matreotype compared with the ECM-High matreotype. Ligand receptor interaction strength was not significantly different for these ligand-receptor pairs between the two matreotypes ZP3-EGFR p=1, ZP3-MERTK p=0.36 Mann-Whitney U-test.

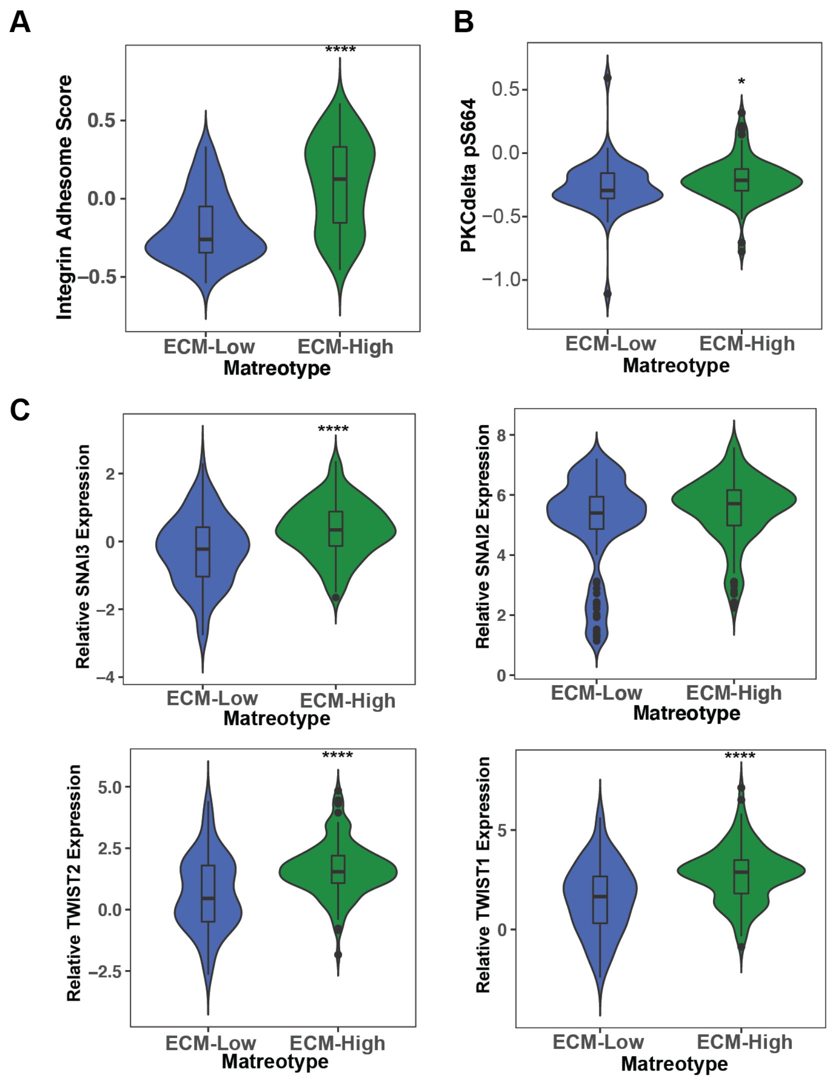

Additional File 1: Figure S8. ECM-driven integrin signaling is associated with EMT and fibroblast activation in the ECM-High matreotype. A) Expression of the integrin adhesome in ECM-High and ECM -Low matreotypes from TCGA RNAseq data. **B)** Expression of integrin phospho-adhesome component pPKCdelta in the ECM-High and ECM-Low matreotypes in TCGA RPPA proteomic data. **C)** Expression of master regulators of EMT and myofibroblast transdifferentiation SNAI2 (p=5.839E-2), SNAI3 (p=5.3E-6), TWIST1 (p=8.4E-8) and TWIST2 (p=4.2E-7) in ECM-High (green) and ECM-Low (blue) matreotypes. Mann-Whitney U-test.

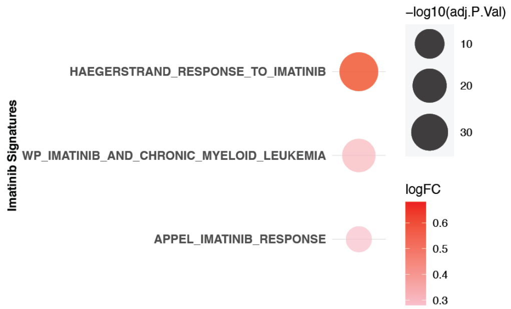

Additional File 1: Figure S9. The poor prognosis matreotype overlaps with ECM remodeling in Idiopathic Pulmonary Fibrosis. A) ECM-High matreotype is enriched in imatinib-response signatures compared with the ECM-Low matreotype. Circle diameter indicates the -$\log_{10}$(p-value), color reflects the log fold change (logFC).

Additional Tables

Additional File 1: Table S1. Matrix Risk Score

Gene	Name	Coefficient	OR [95% CI]	Test data TCGA cohort ROC AUC	NCI-MD ROC AUC	Carcinoma in situ Lesions ROC AUC	Presence in LUAD Matrix Risk Signature
MFAP4	Microfibril associated protein 4	-12.70	3.06E-06 [1.54E-14-6.85E-03]	1.0	0.85	0.72	Yes
RSPO1	R-spondin homolog (Xenopus laevis)	-8.00	3.37E-04 [2.57E-11-0.0325]	1.0	0.84	0.84	Yes
SVEP1	sushi, von Willebrand factor type A, EGF and pentraxin domain containing 1	-7.78	4.17E-04 [1.40E-10-0.0223]	1.0	0.83	0.65	No
FBLN5	Fibulin 5	-7.55	5.26E-04 [1.86E-07-0.0265]	1.0	0.85	0.56	No
NTN4	Netrin 4	-7.14	7.90E-04 [5.09E-06-0.0146]	1.0	0.85	0.75	Yes
WISP2	WNT1 inducible signaling pathway protein 2	-5.61	3.67E-03 [2.67E-05-0.0390]	1.0	0.86	0.63	No
VWF	Von Willebrand Factor	-5.30	4.98E-03 [1.40E-05-0.0511]	1.0	0.84	0.66	No
TNXB	Tenascin XB	-5.27	5.15E-03 [1.13E-06-0.0648]	1.0	0.90	0.71	No
SLIT2	slit homolog 2 (Drosophila)	-5.00	6.73E-03 [2.43E-04-0.0481]	1.0	0.87	-	No
MMRN2	Multimerin 2	-4.87	7.69E-03 [9.44E-05-0.0555]	0.98	0.83	0.47	Yes
LAMB2	Laminin, beta 2	-4.56	0.0105 [7.65E-04-0.0550]	0.99	0.83	0.65	No
CRIM1	cysteine rich transmembrane BMP regulator 1 (chordin-like)	-4.39	0.0124 [1.20E-03-0.0595]	0.99	0.87	0.66	No
GLDN	Gliomedin	-3.91	0.0201 [1.99E-03 - 0.0790]	0.99	0.83	0.67	Yes
ABI3BP	ABI family, member 3 (NESH) binding protein	-3.86	0.0210 [2.34E-03 - 0.0809]	1.0	0.85	-	Yes
MMRN1	Multimerin 1	-3.78	0.0228 [3.09E-03-0.0829]	0.98	0.78	0.81	No
EMILIN2	elastin microfibril interfacer 2	-3.03	0.0484 [0.0124-0.131]	1.0	0.64	0.46	Yes
SPARCL1	SPARC-like 1 (hevin)	-2.89	5.53E-02 [1.28E-02-1.54E-01]	0.91	0.82	0.75	No
LGI4	LGI4	-2.88	0.0563 [0.0146-	1.0	0.79	0.65	Yes

			0.142]				
RSPO2	R-spondin 2 homolog (Xenopus laevis)	-2.87	0.0569 [0.0149-0.147]	1.0	0.87	0.54	No
PRG4	p53-responsive gene 4	-2.75	0.0640 [0.0137-0.168]	0.98	0.86	0.75	No
SPOCK2	sparc/osteonectin, cwcv and kazal-like domains proteoglycan (testican) 2	-2.25	0.106 [0.0439 - 0.205]	1.0	0.81	0.57	Yes
TNR	tenascin R (restrictin, janusin)	-1.55	0.212 [0.11-0.351]	0.93	0.74	0.67	No
CILP2	cartilage intermediate layer protein 2	1.37	3.95 [2.31-7.36]	0.98	0.86	-	Yes
COL10A1	collagen type X alpha 1 chain	1.65	5.21 [2.94-10.5]	0.99	0.85	0.78	Yes
CTHRC1	Collagen triple helix containing protein 1	1.77	5.89 [3.3-12.2]	1.0	0.87	0.83	Yes
SPP1	Secreted phosphoprotein 1/osteopontin	2.56	12.9 [5.59-42.1]	0.98	0.90	0.82	Yes
COL11A1	Collagen type XI alpha 1 chain	2.71	15.0 [6.06-52.5]	1.0	0.89	0.53	Yes
COL7A1	Collagen type VII alpha 1 chain	2.81	16.6 [6.18-79.3]	1.0	0.88	0.69	No

Additional File 1: Table S2. Pan-Cancer Risk Score Results

**Tumor Type Abbreviation**	**Tumor Type Name**	**Tumor vs Non-Tumor Mann Whitney U-test Adjusted P-value**	**ROC Analysis Area Under the Curve**
CESC	Cervical squamous cell carcinoma and endocervical adenocarcinoma	**0.0038**	1.00
LUSC	Lung Squamous NSCLC	**4.7E-31**	0.99
LUAD	Lung Adenocarcinoma	**6.2E-35**	0.99
UCEC	Uterine corpus endometrial carcinoma	**2.9E-08**	0.99
BRCA	Breast invasive carcinoma	**8.5E-62**	0.98
READ	Rectum adenocarcinoma	**1.2E-06**	0.98
BLCA	Bladder urothelial carcinoma	**5.7E-12**	0.98
KIRP	Kidney renal papillary cell carcinoma	**1.7E-17**	0.97
COAD	Colon adenocarcinoma	**2.7E-21**	0.97
KICH	Kidney chromophobe	**8.4E-11**	0.95
PCPG	Pheochromocytoma and paraganglioma	**0.012**	0.94
ESCA	Esophageal carcinoma	**0.00022**	0.93
THYM	Thymoma	**0.047**	0.92
STAD	Stomach adenocarcinoma	**1.6E-08**	0.90
LIHC	Liver hepatocellular carcinoma	**9.6E-15**	0.84
THCA	Thyroid carcinoma	**2.2E-17**	0.84
HNSC	Head and Neck squamous cell carcinoma	**1.9E-12**	0.83
CHOL	Cholangiocarcinoma	**0.038**	0.73
KIRC	Kidney renal clear cell carcinoma	**3.0E-09**	0.72
PRAD	Prostate adenocarcinoma	**4.3E-07**	0.72
PAAD	Pancreatic adenocarcinoma	0.28	0.66
SARC	Sarcoma	0.62	0.60

Additional File 1: Table S3. Optimized Minimum Matrisomal Linear Model for Premalignant Progression

Parameter	Coefficient
Intercept	0.142
RSPO1	-0.332
CTHRC1	0.188
SPP1	0.677
MMRN1	-0.458
COL10A1	0.294
PRG4	-0.328

Additional File 1: Table S4. Clinicodemographic Features of the TCGA Matreotypes

		All	ECM-Low	ECM - High	P-value
**n**		223	94	129	
**Age**		67.5±8.4	65.97±9.39	68.7±7.5	**0.033***
**Gender**	**M**	164 (73.5%)	70 (74.5%)	94 (72.9%)	0.88
	**F**	59 (26.5%)	24 (25.5%)	35 (27.1%)	
**Race**	**Asian**	5 (22.2%)	1 (1.1%)	4 (3.1%)	0.48
	**African American**	10 (4.5%)	3 (3.2%)	7 (5.4%)	
	**White**	146 (65.5%)	63 (67%)	83 (64.3%)	
	**Unknown**	62 (27.8%)	27 (28.7%)	35 (27.1%)	
**Stage**	**IA**	39 (17.5%)	18 (19.2%)	21 (16.3%)	0.88
	**IB**	79 (35.4%)	31 (33.0%)	48 (37.2%)	
	**II**	1 (0.5%)	0 (0%)	1 (0.8%)	
	**IIA**	18 (8.1%)	7 (7.5%)	11 (8.5%)	
	**IIB**	37 (16.6%)	14 (13.9%)	23 (17.8%)	
	**IIIA**	31 (13.9%)	16 (17.0%)	15 (11.6%)	
	**IIIB**	12 (5.4%)	6 (6.4%)	6 (4.7%)	
	**IV**	4 (1.8%)	1 (1.1%)	3 (2.3%)	
	**Unknown**	2 (0.9%)	1 (1.1%)	1 (0.8%)	
**Smoking Status**	**Lifelong Non-smoker**	9 (4.0%)	7 (7.5%)	2 (1.6%)	**0.001**
	**Current Smoker**	34 (15.3%)	20 (21.3%)	14 (10.9%)	
	**Current**	46	10 (10.6%)	36	

	**Reformed Smoker>15yrs**	(20.6%)		(27.9%)	
	**Current Reformed Smoker <=15 yrs**	128 (57.4%)	54 (57.5%)	74 (57.4%)	
	**Current reformed smoker (duration not specified)**	0 (0%)	0 (0%)	0 (0%)	
	**Smoking history not documented**	6 (2.7%)	3 (3.2%)	3 (2.3%)	
**Pack Years**		52.9±33.5	50.6±22.5	54.3±39.1	0.44*
**FGFR-amplification**	**WT**	181	73	108	0.194
	**Gain**	40	19	21	
	**Loss**	2	2	0	

Fishers exact test p-value unless otherwise specified; * Mann-Whitney U-test.

Additional File 1: Table S5. Clinicodemographic Features of the NCI-MD cohort

		**All**	**ECM-Low**	**ECM - High**	**P-value**
**n**		30	14	16	
**Age**		66.4±10.7	69.96±9.0	64.0.7±11.6	0.25*
**Gender**	**M**	22 (73.3%)	9 (64.3%)	13 (81.3%)	0.67
	**F**	7 (23.3%)	4 (28.6%)	3 (18.8%)	
	**Unknown**	1 (3.3%)	1 (7.1%)	0 (0%)	
**Race**	**Hispanic**	1 (3.3%)	1 (7.1%)	0 (0%)	0.11
	**African American**	16 (53.3%)	9 (64.3%)	7 (43.8%)	
	**White**	12 (40%)	3 (21.4%)	9 (56.3%)	
	**Unknown**	1 (3.3%)	1 (7.1%)	0 (0%)	
**Stage**	**I**	21 (70%)	11 (78.6%)	10 (62.5%)	0.36
	**II**	6 (20%)	3 (21.4%)	3 (18.8%)	
	**III**	3 (10%)	0 (0%)	3 (18.8%)	

Fishers exact test p-value unless otherwise specified; * Mann-Whitney U-test.

Additional File 1: Table S6. Clinicodemographic Features of the Tissue Microarray Cohort.

		All	Picrosirius Red Low	Picrosirius Red High	P-value
**n**		94	56	38	
**Age**		65.7±7.9	65.2±8.4	66.4±7.3	0.40*
**Gender**	**M**	66 (70.2%)	39 (69.6%)	27 (71.1%)	1.0
	**F**	28 (29.8%)	17 (30.4%)	11 (28.9%)	
	**Unknown**				
**Stage**	**IA**	11 (11.7%)	6 (10.7%)	5 (13.2%)	0.97
	**IB**	23 (24.5%)	15 (26.8%)	8 (21.1%)	
	**IIA**	42 (44.7%)	24 (42.9%)	18 (47.4%)	
	**IIB**	12 (12.8%)	8 (14.3%)	4 (10.5%)	
	**IIIA**	5 (5.3%)	3 (5.4%)	2 (5.3%)	
	**Unknown**	1 (1.1%)	0 (0%)	1 (2.6%)	
**Smoking History**	**Current Smoker**	6 (6.4%)	3 (5.4%)	3 (7.9%)	0.75
	**Ex-Smoker**	67 (71.3%)	42 (75.0%)	25 (65.8%)	
	**Unknown**	21 (22.3%)	11 (19.6%)	10 (26.3%)	

*Fisher's exact test unless otherwise specified; * Mann-Whitney U-test

Additional File 1: Table S7. Cox Proportional Hazards Model Analysis of Picrosirius Red stained TMAs.

	Model	Parameter	HR [95% CI]	P-value
**All Stages**	*Univariate*	Picrosirius Red stain	1.7 [1.04-2.9]	0.034
	Multivariate	Picrosirius Red Stain	1.9 [1.1-3.2]	0.023
		Stage IA	reference	
		Stage IB	1.4 [0.42-4.3]	0.61
		Stage IIA	1.5 [0.45-5.0]	0.51
		Stage IIB	2.8 [0.84-9.3]	0.094
		Stage IIIA	3.7[0.83-16.7]	0.087
**Early Stages (I and IIA)**	*Univariate*	Picrosirius Red Area	2.4 [1.3-4.4]	0.0051
	Multivariate	Picrosirius Red Area	2.4 [1.3-4.4]	0.0051
		Stage IA	Reference	
		Stage IB	1.4 [0.43-4.4]	0.59
		Stage IIA	2.4 [0.85-6.9]	0.10

Additional File 1: Table S8. Pan-Cancer Prognostic Matreotype Results.

**Tumor Type Abbreviation**	**Tumor Type Name**	**Disease-Specific Survival HR [95% CI]**	**Disease-Specific Survival P-value**	**Progression-Free Survival HR [95% CI]**	**Progression-Free Survival P-value**
ACC	Adrenocortical Carcinoma	1.32 [0.59-2.96]	0.51	1.26 [0.66-2.42]	0.48
BLCA	Bladder Urothelial Carcinoma	1.62 [1.10-2.4]	**0.015**	1.23 [0.90-1.68]	0.19
BRCA	Breast Invasive Carcinoma	1.56 [0.98-2.51]	0.18	1.28 [0.90-1.81]	0.39
CESC	Cervical squamous cell carcinoma and endocervical adenocarcinoma	0.94 [0.53-1.65]	0.83	1.14 [0.70-1.87]	0.60
CHOL	Cholangiocarcinoma	1.07 [0.40-2.87]	0.90	0.91[0.38-2.22]	0.84
COAD	Colon Adenocarcinoma	2.72[1.22-6.06]	**0.014**	1.61 [1.01-2.55]	**0.043**
DLBC	Lymphoid Neoplasm Diffuse Large B-Cell Lymphoma	0.24 [0.02-2.28]	0.21	0.09 [0.01-0.71]	**0.023**
ESCA	Esophageal Carcinoma	1.09 [0.48-2.45]	0.98	1.11 [0.60-2.04]	0.94
GBM	Glioblastoma	1.36 [0.89-2.08]	0.15	1.29 [0.87-1.91]	0.20
HNSC	Head and Neck Squamous Cell Carcinoma	0.94 [0.66-1.35]	0.73	0.83 [0.62-1.11]	0.40
KICH	Kidney Chromophobe	1.74 [0.34-9]	0.51	3.48 [0.75-16.10]	0.11
KIRC	Kidney Renal Clear Cell Carcinoma	1.36 [0.91-2.04]	0.13	1.40 [1.00-1.95]	**0.049**
KIRP	Kidney Renal Papillary Cell Carcinoma	4.08 [1.55-10.73]	**0.0040**	1.99 [1.14-3.49]	**0.016**
LGG	Low-Grade Glioma	2.07 [1.31-3.25]	**0.0020**	1.61 [1.17-2.22]	**0.0040**
LIHC	Liver Hepatocellular Carcinoma	0.88 [0.56-1.37]	0.56	0.77 [0.57-1.03]	0.075
LUAD	Lung Adenocarcinoma	0.89 [0.61-1.31]	0.22	0.97 [0.73-1.29]	0.39
MESO	Head and Neck Squamous Cell Carcinoma	2.30 [1.20-4.39]	**0.042**	1.44 [0.82-2.54]	0.44
OV	Ovarian Serous Adenocarcinoma	1.15 [0.76-1.75]	0.80	1.17 [0.82-1.68]	0.68
PAAD	Pancreatic adenocarcinoma	1.69 [1.00-2.84]	**0.049**	1.54 [1.00-2.37]	**0.050**
PCPG	Pheochromocytoma and paraganglioma	0.76 [0.1-5.60]	0.79	0.90 [0.37-2.21]	0.82
PRAD	Prostate adenocarcinoma	0.51 [0.08-3.23]	0.47	1.17 [0.75-1.83]	0.49
READ	Rectum Adenocarcinoma	0.96 [0.16-5.77]	0.97	1.61 [0.61-4.20]	0.33

SARC	Sarcoma	0.97 [0.62-1.52]	0.89	1.11 [0.78-1.58]	0.55
SKCM	Skin Cutaneous Melanoma	1.71 [0.66-4.40]	0.27	1.23 [0.62-2.41]	0.56
STAD	Stomach adenocarcinoma	1.11 [0.66-1.85]	0.91	1.11 [0.71-1.74]	0.78
THCA	Thyroid carcinoma	0.49 [0.11-2.21]	0.36	0.87 [0.5-1.51]	0.61
THYM	Thymoma	1.04[0.14-7.57]	0.97	1.84 [0.74-4.59]	0.19
UCEC	Uterine corpus endometrial carcinoma	2.00 [0.82-4.85]	0.13	0.96 [0.52-1.78]	0.90
UCS	Uterine Carcinosarcoma	0.87[0.41-1.84]	0.71	0.99 [0.51-1.92]	0.97
UVM	Uveal Melanoma	1.82 [0.70-4.71]	0.22	2.13 [0.92-4.93]	0.079

Data supplied as Additional File 2 (Excel Spreadsheets, .xlsx format)

Additional File 2: Table S1. Differentially Expressed Hallmark Pathways in ECM-High vs ECM-Low Tumors.

Additional File 2: Table S2. Differentially Expressed MSigDb C2 Pathways in ECM-High vs ECM-Low Tumors.

Additional File 2: Table S3. Ligand-Receptor Interactions in ECM-High vs ECM-Low Tumors.

References

1. Chaisaingmongkol J, Budhu A, Dang H, Rabibhadana S, Pupacdi B, Kwon SM, et al. Common molecular subtypes among asian hepatocellular carcinoma and cholangiocarcinoma. Cancer Cell. 2017 Jul 10;32(1):57–70.e3.
2. Parker AL, Toulabi L, Oike T, Kanke Y, Patel D, Tada T, et al. Creatine riboside is a cancer cell-derived metabolite associated with arginine auxotrophy. J Clin Invest. 2022 Jul 15;132(14):e157210.
3. Andrews S. FastQC: A Quality Control Tool for High Throughput Sequence Data. [Internet]. [cited 2019 Aug 12]. Available from: Available online at: http://www.bioinformatics.babraham.ac.uk/projects/fastqc/
4. Wingett SW, Andrews S. FastQ Screen: A tool for multi-genome mapping and quality control. F1000Res. 2018 Aug 24;7:1338.
5. Daley T, Smith AD. Predicting the molecular complexity of sequencing libraries. Nat Methods. 2013 Apr;10(4):325–7.
6. Wang L, Wang S, Li W. RSeQC: quality control of RNA-seq experiments. Bioinformatics. 2012 Aug 15;28(16):2184–5.
7. Okonechnikov K, Conesa A, García-Alcalde F. Qualimap 2: advanced multi-sample quality control for high-throughput sequencing data. Bioinformatics. 2016 Jan 15;32(2):292–4.
8. Martin M. Cutadapt removes adapter sequences from high-throughput sequencing reads. EMBnet j. 2011 May 2;17(1):10.
9. Dobin A, Davis CA, Schlesinger F, Drenkow J, Zaleski C, Jha S, et al. STAR: ultrafast universal RNA-seq aligner. Bioinformatics. 2013 Jan 1;29(1):15–21.
10. Harrow J, Frankish A, Gonzalez JM, Tapanari E, Diekhans M, Kokocinski F, et al. GENCODE: the reference human genome annotation for The ENCODE Project. Genome Res. 2012 Sep;22(9):1760–74.
11. Li B, Dewey CN. RSEM: accurate transcript quantification from RNA-Seq data with or without a reference genome. BMC Bioinformatics. 2011 Aug 4;12:323.
12. Leek JT, Johnson WE, Parker HS, Jaffe AE, Storey JD. The sva package for removing batch effects and other unwanted variation in high-throughput experiments. Bioinformatics. 2012 Mar 15;28(6):882–3.
13. McCarthy DJ, Chen Y, Smyth GK. Differential expression analysis of multifactor RNA-Seq experiments with respect to biological variation. Nucleic

Acids Res. 2012 May;40(10):4288–97.
14. Naba A, Clauser KR, Hoersch S, Liu H, Carr SA, Hynes RO. The matrisome: in silico definition and in vivo characterization by proteomics of normal and tumor extracellular matrices. Mol Cell Proteomics. 2012 Apr;11(4):M111.014647.
15. Durinck S, Spellman PT, Birney E, Huber W. Mapping identifiers for the integration of genomic datasets with the R/Bioconductor package biomaRt. Nat Protoc. 2009 Jul 23;4(8):1184–91.
16. Ritchie ME, Phipson B, Wu D, Hu Y, Law CW, Shi W, et al. limma powers differential expression analyses for RNA-sequencing and microarray studies. Nucleic Acids Res. 2015 Apr 20;43(7):e47.
17. Gu Z, Eils R, Schlesner M. Complex heatmaps reveal patterns and correlations in multidimensional genomic data. Bioinformatics. 2016 Sep 15;32(18):2847–9.
18. Friedman J, Hastie T, Tibshirani R. Regularization Paths for Generalized Linear Models via Coordinate Descent. J Stat Softw. 2010 Feb 21;33(1):1-22.
19. John CR, Watson D, Russ D, Goldmann K, Ehrenstein M, Pitzalis C, et al. M3C: Monte Carlo reference-based consensus clustering. Sci Rep. 2020 Feb 4;10(1):1816.
20. Hänzelmann S, Castelo R, Guinney J. GSVA: gene set variation analysis for microarray and RNA-seq data. BMC Bioinformatics. 2013 Jan 16;14:7.
21. Ramilowski JA, Goldberg T, Harshbarger J, Kloppmann E, Lizio M, Satagopam VP, et al. A draft network of ligand-receptor-mediated multicellular signalling in human. Nat Commun. 2015 Jul 22;6:7866.
22. Wu SZ, Roden DL, Wang C, Holliday H, Harvey K, Cazet AS, et al. Stromal cell diversity associated with immune evasion in human triple-negative breast cancer. EMBO J. 2020 Oct 1;39(19):e104063.
23. Gu Z, Gu L, Eils R, Schlesner M, Brors B. circlize Implements and enhances circular visualization in R. Bioinformatics. 2014 Oct;30(19):2811–2.
24. Lambrechts D, Wauters E, Boeckx B, Aibar S, Nittner D, Burton O, et al. Phenotype molding of stromal cells in the lung tumor microenvironment. Nat Med. 2018 Aug;24(8):1277–89.
25. Faruki H, Mayhew GM, Serody JS, Hayes DN, Perou CM, Lai-Goldman M. Lung adenocarcinoma and squamous cell carcinoma gene expression subtypes demonstrate significant differences in tumor immune landscape. J Thorac Oncol. 2017 Jun;12(6):943–53.

26. Qian J, Olbrecht S, Boeckx B, Vos H, Laoui D, Etlioglu E, et al. A pan-cancer blueprint of the heterogeneous tumor microenvironment revealed by single-cell profiling. Cell Res. 2020 Sep;30(9):745–62.
27. McDonough JE, Ahangari F, Li Q, Jain S, Verleden SE, Herazo-Maya J, et al. Transcriptional regulatory model of fibrosis progression in the human lung. JCI Insight. 2019 Nov 14;4(22):e131597.
28. Keenan AB, Torre D, Lachmann A, Leong AK, Wojciechowicz ML, Utti V, et al. ChEA3: transcription factor enrichment analysis by orthogonal omics integration. Nucleic Acids Res. 2019 Jul 2;47(W1):W212–24.
29. Vennin C, Chin VT, Warren SC, Lucas MC, Herrmann D, Magenau A, et al. Transient tissue priming via ROCK inhibition uncouples pancreatic cancer progression, sensitivity to chemotherapy, and metastasis. Sci Transl Med. 2017 Apr 5;9(384):eaai8504.
30. Vennin C, Mélénec P, Rouet R, Nobis M, Cazet AS, Murphy KJ, et al. CAF hierarchy driven by pancreatic cancer cell p53-status creates a pro-metastatic and chemoresistant environment via perlecan. Nat Commun. 2019 Dec;10(1):3637.
